# Comparison of different reliability estimation methods for single-item assessment: a simulation study

**DOI:** 10.3389/fpsyg.2024.1482016

**Published:** 2024-11-01

**Authors:** Sijun Zhang, Kimberly Colvin

**Affiliations:** ^1^Institute of Educational Sciences, Hunan University, Changsha, China; ^2^School of Education, University at Albany, Albany, NY, United States

**Keywords:** single-item assessment, reliability, simulation study, correction for attenuation, factor analysis, double monotonicity model, Guttman’s λ6, latent class model

## Abstract

Single-item assessments have recently become popular in various fields, and researchers have developed methods for estimating the reliability of single-item assessments, some based on factor analysis and correction for attenuation, and others using the double monotonicity model, Guttman’s λ_6_, or the latent class model. However, no empirical study has investigated which method best estimates the reliability of single-item assessments. This study investigated this question using a simulation study. To represent assessments as they are found in practice, the simulation study varied several aspects: the item discrimination parameter, the test length of the multi-item assessment of the same construct, the sample size, and the correlation between the single-item assessment and the multi-item assessment of the same construct. The results suggest that by using the method based on the double monotonicity model and the method based on correction for attenuation simultaneously, researchers can obtain the most precise estimate of the range of reliability of a single-item assessment in 94.44% of cases. The test length of a multi-item assessment of the same construct, the item discrimination parameter, the sample size, and the correlation between the single-item assessment and the multi-item assessment of the same construct did not influence the choice of method choice.

## 1 Introduction

Reliability of assessment refers to the degree to which an assessment produces stable and consistent results. Three classic methods of estimating reliability are test–retest reliability, parallel-forms reliability, and internal consistency; in practice, internal consistency is most commonly used. Internal consistency assesses the correlation between multiple items in an assessment that are intended to measure the same construct; it is positively affected by increasing test length (Christmann and Aelst, 2006; Tang et al., 2014), so there has often been a drive to develop longer assessments. Despite the positive relationship between internal consistency and test length, short versions of assessments, as well as the extreme single-item assessment, have recently become increasingly popular. Single-item assessments contain only one item to measure a construct. Single-item assessments are sometimes used in educational psychology to assess, for example, STEM identity (McDonald et al., 2019) and subjective academic achievement (Leung and Xu, 2013). They are also found in organizational psychology for selection and assessment of job satisfaction (Robertson and Kee, 2017) and burnout level (Dolan et al., 2015). Furthermore, single-item assessments are used in clinical research to measure depression (Netemyer et al., 2002), life satisfaction (Jovanovic, 2016), happiness (Lukoševičiūtė et al., 2022), and side effects of cancer therapy (Pearman et al., 2018). Single-item assessments can also be found in marketing research in advertising and brand attitude studies (Bergkvist and Rossiter, 2007; Moussa, 2021).

Previous studies have not reached a consensus on whether a single-item assessment is as reliable as the corresponding multi-item assessment. Most researchers agreed that longer assessments are more reliable than shorter assessments; in particular, single-item assessments are considered to be extremely unreliable (Nunnally and Bernstein, 1994; Spector, 1992). Researchers have suggested that with multiple items, random error is more likely to be canceled out by the summation of the item scores into a total score, whereas single-item assessments are more susceptible to random error because the random error of a single item cannot be smoothed out (Mackenzie, 2001; Ryan et al., 1995). On the other hand, Drolet and Morrison (2001) and Dujardin et al. (2021) argued that long tests do not outperform their corresponding shortened (or single-item) versions in terms of reliability. Drolet and Morrison (2001) found evidence that while additional items can significantly increase the correlation of the error term, the incremental information provided by each additional test item is extremely small. In particular, when items are semantically similar, subjects tend to assume that the items are almost the same without reading them carefully so that subjects would make inferences from the content of one item to the remaining items in the test (Allen et al., 2022). Podsakoff et al. (2003) argued that subjects who are exposed to more items tend to discriminate less between them, with earlier items having a strong influence on later items, more items can lead to mindless response behavior, and this mindless response behavior makes long tests even less reliable than their corresponding single-item assessments.

As noted above, there is no consensus on whether a single-item assessment is as reliable as its corresponding multi-item assessment. To investigate whether single-item assessments are reliable, it is necessary to estimate the reliability of single-item assessments. Of the three classic methods for estimating reliability, internal consistency is inappropriate for estimating the reliability of single-item assessments because there is only one item. Test–retest reliability is also inappropriate for single-item assessments measuring transient constructs such as emotions or attitudes, and test–retest reliability is not a perfect choice even for single-item assessments measuring stable constructs because of the practice effect, which may be more severe than for multi-item assessments (Tehan and Tolan, 2007). Only parallel-form reliability is suitable for estimating the reliability of single-item assessments. Based on parallel-form reliability, researchers have proposed five methods for estimating the reliability of single-item assessments: a method based on correction for attenuation (CA), a method that uses factor analysis (FA), a method based on double monotonicity modeling (DMM), a method based on Guttman’s λ_6_, and a method that uses latent class modeling (LCM). These methods use different approximations of the joint cumulative probability. However, no empirical study has investigated which method estimates the reliability of single-item assessment most precisely. Therefore this study uses a simulation study to compare the five methods.

## 2 Methods for estimating the reliability of a single-item assessment

### 2.1 Method CA—correction for attenuation

Method CA is based on the CA formula (Equation 1) from classical test theory (Nunnally, 1967):


(1) $$\rho_{xy}=\frac{r_{xy}}{\sqrt{r_{xx}\cdot r_{yy}}}$$

where $\rho_{xy}$ is the true correlation between the constructs *x* and *y*, $r_{xy}$ is the observed correlation between the two measures, and $r_{xx}$ and $r_{yy}$ are the reliabilities of the two measures. This formula can be used to relate two measures of the same construct, rather than different constructs (Wanous et al., 1997). If two instruments measure the same construct, then the true correlation should be 1.0; replacing $\rho_{xy}$ with 1, using the observed correlation between the single-item assessment and a multi-item assessment of the same construct ( $r_{xy}$ ), and the reliability of the multi-item assessment, it is possible to solve for the reliability of the single-item assessment.

Method CA is the most commonly used method for estimating the reliability of single-item assessments (Zijlmans et al., 2018). However, in practice, the true correlation between a single-item assessment and a multi-item assessment of the same construct ( $\rho_{xy}$ ) is usually less than 1.0, which may lead to an underestimation of the reliability of the single-item assessment (Christmann and Aelst, 2006).

### 2.2 Method FA—factor analysis

Method FA is based on the context of FA where the variance of an item is equal to its communality, specificity, and unreliability, and the reliability of an item is the sum of its communality and specificity (Bailey and Guertin, 1970; Harman, 1976). By conservatively assuming that specificity is zero, the minimum reliability of a single item can thus be estimated by its communality (Ginns and Barrie, 2004). Principal axis factoring is performed on the set of items including those from the multi-item assessment and the single-item assessment of the same construct. Using this technique to estimate reliability for single-item assessments results in underestimation (Arvey et al., 1992; Ginns and Barrie, 2004; Wanous and Hudy, 2001).

### 2.3 Method DMM—double monotonicity model

Method DMM is based on the DMM of Molenaar and Sijtsma (1988). Let us assume that a scale has *N* items (*N* > 1), where *i* and *j* denote items in the scale. Let us further assume that each of these *N* items has *m* + 1 item scores, e.g., if these *N* items are dichotomous, the item scores can be 0 and 1 (*m* = 1); if these *N* items use a 5-point Likert scale, the item scores can be 0, 1, 2, 3, and 4 (*m* = 4). The notation *x* denotes the item *i*’s score, and *y* denotes the item *j*’s score (*x* = 0, …, *m*; *y* = 0, …, *m*). *π*_x(i)_ = *P*(X_i_ ≥ x) denotes the marginal cumulative probability of getting at least *x* on item *i*, *π*_y(j)_ = *P*(X_j_ ≥ y) denotes the marginal cumulative probability of getting at least *y* on item *j*. Obviously, π_0(i)_ = 1 and π_0(j)_ = 1. π_x(i)y(j)_ = *P*(X_i_ ≥ x, X_j_ ≥ y) denotes the joint cumulative probability of obtaining at least *x* on item *i* and at least *y* on item *j*.

If we test item *i* twice independently in the same group of subjects, denoted by *i* and *i*’, then π_x(i)y(i’)_ represents the joint cumulative probability of getting at least *x* on the first test and at least *y* on the second test. In practice, however, we cannot test an item twice independently in the same group of subjects because of the practice effect, so we have to estimate π_x(i)y(i’)_ from a single test.

Reliability is defined as Equation 2


(2) $$\rho xx=\frac{\sigma_{T}^{2}}{\sigma_{X}^{2}}$$

Molenaar and Sijtsma (1988) proved that the true score variance (σ^2^_T_) can be expressed as the sum of the differences between the joint cumulative probability and the product of the marginal cumulative probabilities (Equation 3):


(3) $$\sigma 2T=\overset{N}{\underset{i=1}{\sum }}\overset{N}{\underset{j=1}{\sum }}\overset{m}{\underset{x=1}{\sum }}\overset{m}{\underset{y=1}{\sum }}\;\left[\pi_{x\left(i\right)y\left(j\right)}-\pi_{x\left(i\right)}\pi_{y\left(j\right)}\right]$$

Then reliability can be expressed as


(4) $$\rho xx=\frac{\sum_{i=1}^{N}\sum_{j=1}^{N}\sum_{x=1}^{m}\sum_{y=1}^{m}\;\left[\pi_{x\left(i\right)y\left(j\right)}-\pi_{x\left(i\right)}\pi_{y\left(j\right)}\right]}{\sigma_{x}^{2}}$$

Equation 4 can be further decomposed into two parts, in one part, *i* ≠ *j*, in another part, *i* = *j* (i.e., *j* = *i’*):


(5) $$\begin{array}{c} \rho xx=+\frac{\sum \sum_{i\neq j}^{N}\sum_{x=1}^{m}\sum_{y=1}^{m}\left[\pi_{x\left(i\right)y\left(j\right)}-\pi_{x\left(i\right)}\pi_{y\left(j\right)}\right]}{\sigma_{x}^{2}}\\ +\frac{\sum_{i=1}^{N}\sum_{x=1}^{m}\sum_{y=1}^{m}\left[\pi_{x\left(i\right)y\left(i^{\prime }\right)}-\pi_{x\left(i\right)}\pi_{y\left(i\right)}\right]}{\sigma_{x}^{2}} \end{array}$$

Equation 5 can be adapted to estimate the reliability of a single item in a multi-item assessment (Zijlmans et al., 2018), where the first ratio and the first summation sign in the second ratio disappear, the reliability of an item is:


(6) $$\rho ii^{’}=\frac{\sum_{x=1}^{m}\sum_{y=1}^{m}\left[\pi_{x\left(i\right)y\left(i^{\prime }\right)}-\pi_{x\left(i\right)}\pi_{y\left(i\right)}\right]}{\sigma_{x_{i}}^{2}}$$

In Equation 6, in addition to π_x(i)y(i’)_, other terms can be observed or calculated from a single test.

Molenaar and Sijtsma (1988) developed method DMM for estimating π_x(i)y(i’)_ in Equation 6, which they described using an artificial 4-item assessment, where each item has three ordered categories (i.e., item scores can be 0, 1, 2). Table 1 shows the marginal cumulative probabilities for 4 items.

**Table 1:** Marginal cumulative probabilities for 4 items.

	*i* = 1	*i* = 2	*i* = 3	*i* = 4
π_0(i)_	1	1	1	1
π_1(i)_	0.9	0.8	0.7	0.6
π_2(i)_	0.5	0.4	0.3	0.2

We rank all the marginal cumulative probabilities in Table 1 from small to large:


$$\pi_{2\left(4\right)}<\pi_{2\left(3\right)}<\pi_{2\left(2\right)}<\pi_{2\left(1\right)}<\pi_{1\left(4\right)}<\pi_{1\left(3\right)}<\pi_{1\left(2\right)}<\pi_{1\left(1\right)}\text{.}$$

Then construct a matrix (Table 2) of joint cumulative probabilities in which the rows and columns are ordered by the size of the corresponding marginal cumulative probabilities, where NA indicates that a joint cumulative probability is unobservable and must be estimated. For convenience, π_x(1)y(i’)_ is in the cell or row *r* and column *c*; π_x(1)y(i’)_ is denoted *P*_r,c_, and the corresponding marginal cumulative probabilities are *P*_r_ and *P*_c_, respectively.

**Table 2:** Joint cumulative probabilities, π_x(i)y(j)_.

	π_2(4)_	π_2(3)_	π_2(2)_	π_2(1)_	π_1(4)_	π_1(3)_	π_1(2)_	π_1(1)_
π_2(4)_	NA	−0.2	0.2	0.2	NA	0.2	0.2	0.2
π_2(3)_	0.2	NA	0.3	0.3	0.3	NA	0.3	0.3
π_2(2)_	0.2	0.3	NA	0.4	0.4	0.4	NA	0.4
π_2(1)_	0.2	0.3	0.4	NA	0.5	0.5	0.5	NA
π_1(4)_	NA	0.3	0.4	0.5	NA	0.6	0.6	0.6
π_1(3)_	0.2	NA	0.4	0.5	0.6	NA	0.7	0.7
π_1(2)_	0.2	0.3	NA	0.5	0.6	0.7	NA	0.8
π_1(1)_	0.2	0.3	0.4	NA	0.6	0.7	0.8	NA

To calculate NA in Table 2, we define four types of joint cumulative probabilities: (1) the lower neighboring joint cumulative probability: *P*_lo_ = *P*_r + 1, c_; (2) the right neighboring joint cumulative probability: *P*_ri_ = *P*_r,c+_; (3) the upper neighboring joint cumulative probability: *P*_up_ = *P*_r-1,c_; and (4) the left neighboring joint cumulative probability: *P*_le_ = *P*_r, c-1_. Not all four neighboring joint cumulative probabilities exist for each NA, e.g., for *P*_1,5_, *P*_up_ does not exist, *P*_lo_ = 0.3, *P*_le_ = 0.2, and *P*_ri_ = 0.2.

Molenaar and Sijtsma (1988) estimate π_x(i)y(i’)_ (i.e., *P*_r,c_) eight times using the following eight different equations:


(7) $$P\left(1\right)r,c=Plo\frac{P_{r}}{P_{r+1}}$$

(8) $$P\left(2\right)r,c=Pri\frac{P_{c}}{P_{c+1}}$$

(9) $$P\left(3\right)r,c=P\mathrm{up}\frac{P_{r}}{P_{r-1}}$$

(10) $$P\left(4\right)r,c=Ple\frac{P_{c}}{P_{c-1}}$$

(11) $$P\left(5\right)r,c=Plo\frac{1-P_{r}}{1-P_{r+1}}-Pc\frac{P_{r+1}-P_{r}}{1-P_{r+1}}$$

(12) $$P\left(6\right)r,c=Pri\frac{1-P_{c}}{1-P_{c+1}}-Pr\frac{P_{c+1}-P_{c}}{1-P_{c+1}}$$

(13) $$P\left(7\right)r,c=P\mathrm{up}\frac{1-P_{r}}{1-P_{r-1}}-Pc\frac{P_{r}-P_{r-1}}{1-P_{r-1}}$$

(14) $$P\left(8\right)r,c=Ple\frac{1-P_{c}}{1-P_{c-1}}-Pr\frac{P_{c}-P_{c-1}}{1-P_{c-1}}$$

*P*_r,c_ is estimated as the mean of eight estimates in Equations 7–14. For example, using Equations 7–14 to estimate π_2(4)1(4′)_ (i.e., *P*_1,5_) in Table 2, π_2(4)1(4′)_ = 0.21. However, Molenaar and Sijtsma (1988) pointed out that *P*_r,c_ should be in the interval (*P*_r_*P*_c_, min(*P*_r_, *P*_c_)); for π_2(4)1(4′)_, the lower bound is 0.2 × 0.6 = 0.12 and the upper bound is 0.2, so π_2(4)1(4′)_ = 0.2.

Now that π_x(i)y(i’)_ can be estimated, we can use Equation 6 to estimate the reliability of an item in a multi-item scale. To estimate the reliability of a single-item assessment, researchers combine a single-item assessment and a multi-item assessment of the same construct into one scale.

Method DMM is based on the double monotonicity model. This model has two assumptions, first, that the multi-item scale is unidimensional; and second, that there is no intersection of the response functions between different items (Sijtsma and Molenaar, 2002). Therefore, to use method DMM to estimate the reliability of a single-item assessment, the corresponding multi-item assessment should be unidimensional and have non-intersecting item response functions.

### 2.4 Method λ_6_

Method λ_6_ is based on Guttman’s λ_6_ (Guttman, 1945) to estimate π_x(i)y(i’)_ in Equation 6. Let us assume that one scale has *N* items (*N* > 1). We ran a regression to predict the item score *X*_i_ with the remaining *N*-1 item scores (*i* = 1, …, *N*), where ε^2^_i_ denotes the variance of the residual error of the regression. Guttman (1945) defined λ_6_ as the lower limit of the reliability of a multi-item scale:


(15) $$\lambda 6=1-\frac{\sum_{i=1}^{N}\varepsilon_{i}^{2}}{\sigma_{X}^{2}}$$

To calculate ε^2^_i_, let us use Σ_ii_ to represent the (*N*-1) × (*N*-1) variance–covariance matrix for *N*-1 items other than item *i*. *σ*_i_ denotes the (*N*-1) × 1 vector containing the covariances of item *i* with the other *N*-1 items. Jackson and Agunwamba (1977) verified that ε^2^_i_ can be expressed as:


(16) $${\varepsilon^{2}}_{i}={\sigma^{2}}_{\mathrm{Xi}}–{\sigma^{’}}_{i}{\left(\Sigma_{ii}\right)}^{-1}\sigma_{i}$$

To estimate the reliability of an item in a multi-item scale, we insert Equation 16 into Equation 15:


(17) $$\lambda 6i=1-\frac{\sigma_{x_{i}}^{2}-\sigma_{i}^{\prime }{\left(\Sigma_{ii}\right)}^{-1}\sigma_{i}}{\sigma_{x_{i}}^{2}}=\frac{\sigma_{i}^{\prime }{\left(\Sigma_{ii}\right)}^{-1}\sigma_{i}}{\sigma_{x_{i}}^{2}}$$

Since λ_6_ is the lower limit of reliability, Equation 17 can be approximated to Equation 18 by Equation 6 (Zijlmans et al., 2018):


(18) $$\lambda 6i=\frac{\sigma_{i}^{\prime }{\left(\Sigma_{ii}\right)}^{-1}\sigma_{i}}{\sigma_{x_{i}}^{2}}=\frac{\sum_{x=1}^{m}\sum_{y=1}^{m}\left[\pi_{x\left(i\right)y\left(i^{\prime }\right)}-\pi_{x\left(i\right)}\pi_{y\left(i\right)}\right]}{\sigma_{x_{i}}^{2}}$$

π_x(i)y(i’)_ can be expressed as:


(19) $$\pi x\left(i\right)y\left(i^{’}\right)=\frac{\sigma_{i}^{\prime }{\left(\Sigma_{ii}\right)}^{-1}\sigma_{i}}{m^{2}}+\pi x\left(i\right)\pi y\left(i\right)$$

By inserting Equation 19 into Equation 6, we can calculate the reliability of an item in a multi-item scale. To estimate the reliability of a single-item assessment, we again combine a single-item assessment and a multi-item assessment of the same construct into one scale.

Since λ_6_ is the lower limit of reliability, method λ_6_ will underestimate the reliability of single-item assessments in most cases.

### 2.5 Method LCM—latent class modeling

Method LCM uses the latent class model to estimate π_x(i)y(i’)_ in Equation 6 (McCutcheon, 1987; McCutcheon et al., 2002). Let us assume that a group of subjects take an *N*-item survey, each of these *N* items having *m* + 1 item scores. Suppose further that there is a latent categorical variable *ξ* that accounts for the relationship between the *N* items, the latent variable ξ has *Q* latent classes. McCutcheon et al. (2002) defined the latent class model as:


(20) $$P\left(X1=x1,\ldots ,XN=xN\right)=\overset{Q}{\underset{q=1}{\sum }}P\left(\xi =q\right)\overset{N}{\underset{N=1}{\prod }}P\left(X_{i}=x_{i}|\xi =q\right)$$

where *P*(*X*_1_ = *x*_1_, …, *X*_N_ = *x*_N_) is the joint probability distribution of the *N* items, *P*(ξ = *q*) is the probability that a randomly selected subject is in latent class *q* of latent variable ξ, *P*(*X*_i_ = *x*_i_| ξ = *q*) is the conditional probability of a particular item score given class *q*.

The latent class model in Equation 20 asserts that items are conditionally independent given a particular class in ξ (Goodman, 2002). Conditional independence means that when the latent variable ξ that influences subjects’ responses to items is held constant, subjects’ responses to any two items are independent.

Zijlmans et al. (2018) restricted Equation 20 to only one item in a multi-item scale to estimate π_x(i)y(i’)_:


(21) $$\pi x\left(i\right)y\left(i^{’}\right)=\overset{m}{\underset{u=x}{\sum }}\overset{m}{\underset{v=y}{\sum }}\overset{Q}{\underset{q=1}{\sum }}P\left(\xi =q\right)P\left(X_{i}=u|\xi =q\right)P\left(X_{i}=v|\xi =q\right)$$

Method LCM inserts Equation 21 into Equation 6 to estimate the reliability of an item in a multi-item scale. To estimate the reliability of a single-item assessment, we combine a single-item assessment and a multi-item assessment of the same construct into one scale.

Method LCM will accurately estimate the reliability of single-item assessments only if *Q* latent classes are accurately selected and *P*(ξ = *q*), *P*(*X*_i_ = *u*|ξ = *q*), *P*(*X*_i_ = *v*|ξ = *q*) in Equation 21 are equal to the population parameter (McCutcheon et al., 2002). These two assumptions are difficult to meet in reality, so method LCM may often misestimate the reliability of single-item assessments.

### 2.6 Purpose of this study

Of the five methods for estimating the reliability of single-item assessment, researchers do not know which is the best. The most commonly used method is method CA, which assumes that the true correlation between single-item assessments and their corresponding multi-item test is 1. In practice, the true correlation is unlikely to be 1, so method CA often underestimates the reliability of single-item assessments (Krieglstein et al., 2022). Method FA uses the communality of a single item as a conservative estimate of the reliability of single-item assessments, while many studies of single-item assessments have shown that the results via method FA are not much lower than those via method CA (Buchner et al., 2024; Dolan et al., 2015; Leung and Xu, 2013; McDonald et al., 2019). Given that λ_6_ is the lower bound of reliability, method λ_6_ will underestimate the reliability of single-item assessments in most cases. Method DMM and method LCM are based on assumptions that are often violated.

This study plans to conduct a simulation study to investigate which method most accurately estimates the reliability of single-item assessment. The simulation study will generate scores from single-item assessments and item scores from multi-item scales. As Likert scales are the most popular format used in scale design (Foddy, 1994), all simulated items will be polytomous. Given that single-item assessments are typically designed to estimate unidimensional constructs, the multi-item scale in this study will be unidimensional. To fully represent different types of multi-item scales in reality, the multi-item scales in this study will vary in two aspects, test length and discrimination parameters. For the simulated datasets in each condition, this study will compare the median bias, IQR, RMSE, and percentage of outliers (Zijlmans et al., 2018) produced by method CA, method FA, method DMM, method λ_6_, and method LCM for the single-item assessments.

## 3 Method

### 3.1 Data-generating model

Simulation and statistical analyses were performed using R, version 4.3.2, graphs were generated using the package “ggplot2,” and scripts were uploaded as Supplementary material. Method CA, method FA, method DMM, method λ_6_, and method LCM all use a single-item assessment and a multi-item assessment of the same construct to estimate the reliability of the single-item assessment, so in the simulation study, the last item of each simulation dataset is the single-item assessment, other items construct the multi-item assessment of the same construct. For example, a simulation dataset contains scores for 7 items, the first 6 items are items from the multi-item assessment, and the last item is a single-item assessment.

Because single-item assessments are designed to measure unidimensional or global constructs, researchers usually use multiple single-item assessments to measure multidimensional constructs (one item for one dimension), so unidimensional multi-item assessments were used in this simulation study. After reviewing approximately 100 articles on single-item assessments, it was found that all single-item assessments measuring psychological constructs were polytomous, most of which used a 5-point Likert scale. Joshi et al. (2015) found that the 5-point Likert scale was the most commonly used and that 7-point or 10-point Likert scales did not outperform the 5-point Likert scale in terms of psychometric properties (Colvin et al., 2020; Jebb et al., 2021). Therefore, all single-item assessments and multi-item assessments of the same construct in this simulation study would use a 5-point Likert scale.

All polytomous item scores were generated using the multidimensional graded response model (Penfield, 2014):


(22) $$P\left(\mathrm{Xi}\geq x|\theta \right)=\frac{\exp\left[\sum_{q=1}^{Q}a_{iq}\left(\theta_{q}-b_{ix}\right)\right]}{1+\exp\left[\sum_{q=1}^{Q}a_{iq}\left(\theta_{q}-b_{ix}\right)\right]}$$

where θ = (θ_1_, …, θ_Q_) represents the Q-dimensional latent variable vector which has a *Q*-variate standard normal distribution, *P*(*X*_i_ ≥ *x*|θ) denotes the probability that the item score is greater than or equal to *x* for a given value of θ for item *i*, *a_iq_* is the discrimination parameter of item *i*, relative to the latent variable *q*, *b*_ix_ is the difficulty parameter for categorical *x* (*x* = 1, 2, 3, 4) of item *i*.

### 3.2 Simulation design

#### 3.2.1 Design of multi-item assessments of the same construct

The reliability of a single-item assessment is related to the reliability of the multi-item assessment of the same construct, the more reliable the multi-item test, the more reliable the single-item assessment (Wanous and Hudy, 2001). The reliability of the multi-item test is related to the length of the test, so the simulation study would examine the influence of the length of the multi-item scale on the estimate of the reliability of the single-item assessment. By reviewing meta-analyses that examined the correlation between single-item assessments and multi-item assessments of the same construct (Ruekert and Churchill, 1984; Wanous et al., 1997; Wanous and Hudy, 2001), as well as by reviewing approximately 40 articles that examined new single-item assessments via multi-item assessments of the same construct, we found that the test length of multi-item assessments of the same construct ranged from 6 items to 16 items. This simulation study therefore simulated three different lengths of the multi-item assessment: short (6 items), medium (12 items), and long (18 items).

In practice, if a category, *x*, in Equation 22 represents a wide range of θ (e.g., θ ranges from 0 to 8), the difficulty parameter (*b*_ix_) should advance by less than 5 (i.e., *b*_i(x + 1)_ - *b*_ix_ < 5) because the category boundaries are far apart and the middle part of this category loses measurement accuracy. On the other hand, to avoid two adjacent categories representing the same range of θ, *b*_ix_ should advance by at least 1.4 (i.e., *b*_i(x + 1)_–*b*_ix_ ≥ 1.4) (Linacre, 1999). If *b*_adv_ is (*b*_i(x + 1)_–*b*_ix_), then following Linacre (1999) *b*_avd_ should be a random value between 1.4 and 5. However, large *b*_avd_ values are rare in practice; moreover, most items are good at detecting a small range of θ in practice, for example, most items detect θ from −4 to 4 in practice. Very few items are designed to detect extreme θ values (such as 8 or −9) (Chen et al., 2012). To better replicate what happens in reality, the simulation study defined *b*_i1_ as a random value from the interval [−4.2, 0], *b*_i2_ as *b*_i1_ + *b*_avd_, *b*_i3_ as *b*_i2_ + *b*_avd_, *b*_i4_ as *b*_i3_ + *b*_avd_, where *b*_avd_ is a random value from the interval [1.4, 2.5].

According to the item selection study by Chen et al. (2012) and the questionnaire development study by Edelen and Reeve (2007), the range of discrimination parameters for about 560 items is from 0.45 to 2.75. Therefore, in the simulation study, the discrimination parameter, *a*, was defined as a random value chosen from the interval [0.4, 2.8].

Although equally discriminating multi-item assessments, such as those using the Rasch model, have been heavily criticized for reducing the reliability of multi-item scale in most cases (Lord, 1977), in practice both equally and unequally discriminating multi-item assessments are used. Thus, in the simulation study, multi-item assessments differed in the discriminating condition: equally discriminating vs. unequally discriminating. In equally discriminating multi-item assessments, the multidimensional partial credit model is most commonly used to develop equally discriminating polytomous assessments (Masters, 2016; Yao and Schwarz, 2006), while the multidimensional partial credit model is a special case of the multidimensional graded response model in which the discrimination parameters are set to 1, the simulation study defined all item discrimination parameters as 1 for equally discriminating multi-item assessments. In unequally discriminating multi-item assessments, each item’s discrimination parameter is a random value chosen from the interval [0.4, 2.8], the discrimination of one item is not related to the discrimination of other items (Edelen and Reeve, 2007).

In total, there were six types of multi-item assessments of the same construct: short equally discriminating multi-item assessment, short unequally discriminating multi-item assessment, medium equally discriminating multi-item assessment, medium unequally discriminating multi-item assessment, long equally discriminating multi-item assessment, and long unequally discriminating multi-item assessment.

#### 3.2.2 Design of single-item assessments

For single-item assessment, the simulation study defined the discrimination parameter, *a*, as a random value chosen from the interval [0.4, 2.8]; we further defined the first difficulty parameters (*b*_1_) as a random value from the interval [−4.2, 0], *b*_i2_ as *b*_i1_ + *b*_avd_, *b*_i3_ as *b*_i2_ + *b*_avd_, *b*_i4_ as *b*_i3_ + *b*_avd_, where *b*_avd_ was a random value from the interval [1.4, 2.5].

#### 3.2.3 Correlation between single-item assessment and multi-item assessment

Although the single-item assessment and its corresponding multi-item assessment measure the same construct, the correlation between these two assessments is unlikely to be 1.0 in reality (i.e., *Q* = 2 in Equation 22). The meta-analysis by Wanous et al. (1997) reported a mean correlation between single-item assessments and their corresponding multi-item assessments of 0.67 in the context of job satisfaction; another meta-analysis of teaching effectiveness reported mean correlations of 0.84 (Wanous and Hudy, 2001). In this simulation study, three correlations were defined between single-item assessments and their corresponding multi-item assessments: 0.65, 0.75, and 0.85.

#### 3.2.4 Other properties of simulation data

Cho (2024) and Van der Ark et al. (2011) verified that sample size did not affect the difference between estimated reliability and true reliability for multi-item tests through a simulation study, but it is unclear whether sample size affects the difference between estimated and true reliability for single-item assessments. Furthermore, based on Charter’s (1999) study, the width of the confidence interval for estimated reliability is a function of sample size (*N*), with a minimum of 400 subjects recommended for reliability studies. For this simulation study, two sample sizes would be simulated: small (*N* = 400) and large (*N* = 1,000).

In total, there are 36 simulation conditions in this study, as listed in Table 3.

**Table 3:** Definition of 36 simulation conditions.

Condition number	Length of multi-item assessment	Discrimination of multi-item assessment	Correlation	Sample size
1	short	equally	0.65	400
2	medium	equally	0.65	400
3	long	equally	0.65	400
4	short	unequally	0.65	400
5	medium	unequally	0.65	400
6	long	unequally	0.65	400
7	short	equally	0.75	400
8	medium	equally	0.75	400
9	long	equally	0.75	400
10	short	unequally	0.75	400
11	medium	unequally	0.75	400
12	long	unequally	0.75	400
13	short	equally	0.85	400
14	medium	equally	0.85	400
15	long	equally	0.85	400
16	short	unequally	0.85	400
17	medium	unequally	0.85	400
18	long	unequally	0.85	400
19	short	equally	0.65	1,000
20	medium	equally	0.65	1,000
21	long	equally	0.65	1,000
22	short	unequally	0.65	1,000
23	medium	unequally	0.65	1,000
24	long	unequally	0.65	1,000
25	short	equally	0.75	1,000
26	medium	equally	0.75	1,000
27	long	equally	0.75	1,000
28	short	unequally	0.75	1,000
29	medium	unequally	0.75	1,000
30	long	unequally	0.75	1,000
31	short	equally	0.85	1,000
32	medium	equally	0.85	1,000
33	long	equally	0.85	1,000
34	short	unequally	0.85	1,000
35	medium	unequally	0.85	1,000
36	long	unequally	0.85	1,000

Now that the properties of the simulation data are defined, we can generate data sets. For each replication, *N* latent variable vectors, *θ*_1_, θ_2_, …, θ_N_, were randomly drawn from the θ standard normal distribution. For each set of latent variable scores, four cumulative response probabilities were generated for each item using Equation 22, and then item scores were drawn from the multinomial distribution using these four cumulative response probabilities. In each condition, 1,000 data sets were drawn. The studies by Liu et al. (2022) and Trizano-Hermosilla et al. (2021) were referenced in the data generation process.

### 3.3 Statistical analyses

To examine the precision of the five methods for estimating the reliability of single-item assessments, we first need to know the true reliability of single-item assessments (*ρ*_ii_). For single-item assessments, the simulation study generated one million sets of item scores and then we used the variance based on the latent variable vectors (θs) of the 1 million sets of item scores to divide by the variance of the single-item score to obtain the true single-item assessment’s reliability (Feinberg and Rubright, 2016; Zijlmans et al., 2018). As shown in Table 3, some simulation conditions differ only in sample size (e.g., condition 1 and condition 19, condition 2 and condition 20); the true single-item assessments’ reliabilities in these conditions are the same given the calculation process. Second, we estimated the reliability of single-item assessments (*r*_ii_) using method CA, method FA, method DMM, method λ_6_, and method LCM. In each simulation condition, given that 1,000 datasets were drawn, 1,000 estimates of the reliability of single-item assessments were obtained using each method. Given that there are different conditions in the simulation study, a method may be the most precise in one condition, while it may not be the best choice in other conditions. Therefore, in each simulation condition, we used four criteria to examine the precision of the five methods. The first three criteria are based on bias, (*r*_ii_–ρ_ii_), and relative bias, $(\frac{r_{ii}-\rho_{ii}}{\rho_{ii}}$ ):

1. Median of bias and relative bias
2. IQR of bias and relative bias
3. Percentage of outliers of bias and relative bias (they are actually the same), we defined outliers as data less than *Q*1-1.5 × IQR or greater than *Q*3 + 1.5 × IQR (*Q*1 is the first quartile, *Q*3 is the third quartile; Dawson, 2011).

The final criterion is the root mean square error (RMSE). Root mean square error is the standard deviation of the prediction errors, where the prediction errors are a measure of the difference between the predicted value and the true value (Chai and Draxler, 2014). In this simulation study, prediction errors are the absolute value of (*r*_ii_–ρ_ii_)s, and RMSE is the standard deviation of |*r*_ii_–ρ_ii_|s.

## 4 Results

### 4.1 Five methods’ performance in four criteria

For method CA, method FA, method DMM, method λ_6_, and method LCM, the median and IQR of bias, the median and IQR of relative bias, the percentage of outliers of bias (i.e., the percentage of outliers of relative bias), and the RMSE (the standard deviation of|*r*_ii_–ρ_ii_|s) are presented in Tables 4–7.

**Table 4:** Median and IQR (presented in parentheses) of the bias using method CA, method FA, method DMM, method λ_6_, and method LCM in simulation conditions 1–36.

Condition number	Method CA	Method FA	Method DMM	Method λ_6_	Method LCM
1	−0.11 (0.05)	−0.11 (0.05)	−0.04 (0.06)	−0.12 (0.04)	−0.10 (0.05)
2	−0.10 (0.06)	−0.10 (0.06)	−0.07 (0.05)	−0.11 (0.060)	−0.11 (0.07)
3	−0.04 (0.06)	−0.04 (0.06)	−0.01 (0.05)	−0.03 (0.05)	−0.04 (0.06)
4	−0.07 (0.05)	−0.06 (0.05)	0.02 (0.06)	−0.09 (0.04)	−0.06 (0.06)
5	−0.11 (0.05)	−0.11 (0.05)	0.02 (0.06)	−0.10 (0.04)	−0.10 (0.05)
6	−0.11 (0.03)	−0.11 (0.03)	−0.01 (0.04)	−0.07 (0.05)	−0.11 (0.04)
7	−0.08 (0.07)	−0.08 (0.07)	−0.05 (0.05)	−0.13 (0.05)	−0.08 (0.09)
8	0.01 (0.06)	0.01 (0.06)	0.02 (0.06)	−0.01 (0.05)	0.004 (0.06)
9	−0.11 (0.05)	−0.11 (0.05)	−0.07 (0.05)	−0.10 (0.05)	−0.11 (0.05)
10	0.01 (0.06)	0.02 (0.07)	0.07 (0.06)	−0.02 (0.05)	0.02 (0.07)
11	−0.03 (0.05)	−0.03 (0.05)	−0.01 (0.05)	−0.04 (0.04)	−0.04 (0.06)
12	−0.13 (0.07)	−0.13 (0.07)	−0.11 (0.07)	−0.13 (0.07)	−0.13 (0.06)
13	−0.06 (0.06)	−0.06 (0.06)	−0.01 (0.05)	−0.10 (0.04)	−0.06 (0.07)
14	−0.06 (0.06)	−0.07 (0.06)	−0.11 (0.05)	−0.11 (0.06)	−0.08 (0.07)
15	0.02 (0.06)	0.02 (0.06)	0 (0.05)	−0.01 (0.04)	0.02 (0.06)
16	−0.03 (0.05)	−0.03 (0.05)	0.01 (0.05)	−0.04 (0.05)	0.11 (0.06)
17	−0.13 (0.03)	−0.13 (0.03)	−0.03 (0.05)	−0.11 (0.03)	−0.13 (0.04)
18	−0.09 (0.04)	−0.09 (0.04)	0.02 (0.04)	−0.07 (0.05)	−0.09 (0.05)
19	−0.09 (0.03)	−0.09 (0.03)	−0.01 (0.03)	−0.11 (0.02)	−0.08 (0.03)
20	−0.10 (0.03)	−0.10 (0.04)	−0.07 (0.03)	−0.13 (0.03)	−0.10 (0.04)
21	−0.04 (0.03)	−0.04 (0.04)	−0.01 (0.02)	−0.05 (0.04)	−0.04 (0.04)
22	−0.16 (0.03)	−0.16 (0.03)	−0.07 (0.03)	−0.17 (0.03)	−0.15 (0.03)
23	−0.04 (0.05)	−0.04 (0.05)	0.02 (0.06)	−0.04 (0.04)	−0.06 (0.05)
24	−0.16 (0.03)	−0.16 (0.03)	−0.04 (0.06)	−0.10 (0.05)	−0.15 (0.04)
25	−0.09 (0.07)	−0.09 (0.07)	−0.06 (0.05)	−0.14 (0.05)	−0.10 (0.09)
26	0.03 (0.04)	0.03 (0.04)	0.03 (0.03)	−0.01 (0.03)	0.03 (0.04)
27	−0.06 (0.05)	−0.06 (0.05)	−0.02 (0.05)	−0.05 (0.05)	−0.07 (0.05)
28	−0.08 (0.04)	−0.08 (0.04)	−0.05 (0.04)	−0.13 (0.03)	−0.08 (0.04)
29	−0.03 (0.05)	−0.03 (0.05)	−0.01 (0.06)	−0.05 (0.04)	−0.04 (0.06)
30	−0.12 (0.07)	−0.11 (0.07)	−0.10 (0.07)	−0.12 (0.07)	−0.12 (0.06)
31	−0.07 (0.06)	−0.07 (0.06)	−0.01 (0.05)	−0.11 (0.04)	−0.07 (0.07)
32	−0.04 (0.04)	−0.06 (0.04)	−0.11 (0.03)	−0.11 (0.03)	−0.07 (0.04)
33	0.03 (0.06)	0.03 (0.06)	0 (0.05)	−0.03 (0.04)	0.03 (0.06)
34	−0.03 (0.05)	−0.03 (0.05)	−0.01 (0.05)	−0.05 (0.05)	0.12 (0.06)
35	−0.12 (0.03)	−0.12 (0.03)	−0.02 (0.04)	−0.10 (0.03)	−0.12 (0.040)
36	−0.08 (0.04)	−0.08 (0.04)	0.01 (0.04)	−0.07 (0.05)	−0.08 (0.05)

**Table 5:** Median and IQR (presented in parentheses) of the relative bias of method CA, method FA, method DMM, method λ_6_, and method LCM in simulation conditions 1–36.

Condition number	Method CA	Method FA	Method DMM	Method λ_6_	Method LCM
1	−0.22 (0.10)	−0.22 (0.11)	−0.07 (0.11)	−0.25 (0.07)	−0.21 (0.11)
2	−0.20 (0.13)	−0.20 (0.13)	−0.14 (0.10)	−0.22 (0.11)	−0.21 (0.13)
3	−0.08 (0.12)	−0.08 (0.12)	−0.02 (0.10)	−0.06 (−0.10)	−0.09 (0.11)
4	−0.12 (0.09)	−0.12 (0.09)	0.04 (0.10)	−0.16 (0.07)	−0.10 (0.10)
5	−0.19 (0.09)	−0.19 (0.09)	0.03 (0.10)	−0.17 (0.08)	−0.18 (0.09)
6	−0.21 (0.06)	−0.21 (0.06)	−0.01 (0.07)	−0.13 (0.09)	−0.21 (0.07)
7	−0.17 (0.13)	−0.17 (0.14)	−0.10 (0.10)	−0.27 (0.10)	−0.17 (0.19)
8	0.04 (0.21)	0.04 (0.21)	0.07 (0.20)	−0.04 (0.18)	0.02 (0.22)
9	−0.31 (0.16)	−0.32 (0.16)	−0.20 (0.16)	−0.30 (0.15)	−0.32 (0.16)
10	0.05 (0.23)	0.06 (0.25)	0.28 (0.23)	−0.09 (0.18)	0.08 (0.28)
11	−0.06 (0.11)	−0.07 (0.12)	−0.01 (0.12)	−0.10 (0.09)	−0.09 (0.13)
12	−0.34 (0.18)	−0.34 (0.18)	−0.28 (0.19)	−0.34 (0.18)	−0.35 (0.15)
13	−0.12 (0.11)	−0.12 (0.11)	−0.01 (0.09)	−0.19 (0.08)	−0.11 (0.14)
14	−0.14 (0.14)	−0.15 (0.14)	−0.24 (0.10)	−0.23 (0.13)	−0.18 (0.14)
15	0.05 (0.12)	0.05 (0.120)	0 (0.11)	−0.03 (0.09)	0.03 (0.14)
16	−0.04 (0.10)	−0.04 (0.10)	−0.01 (0.10)	−0.07 (0.09)	0.21 (0.11)
17	−0.26 (0.06)	−0.26 (0.06)	−0.05 (0.09)	−0.22 (0.05)	−0.24 (0.07)
18	−0.31 (0.13)	−0.31 (0.13)	0.07 (0.14)	−0.24 (0.16)	−0.29 (0.16)
19	−0.18 (0.06)	−0.18 (0.06)	−0.02 (0.07)	−0.23 (0.04)	−0.17 (0.06)
20	−0.20 (0.07)	−0.20 (0.07)	−0.13 (0.06)	−0.25 (0.06)	−0.20 (0.08)
21	−0.09 (0.07)	−0.09 (0.07)	−0.03 (0.05)	−0.11 (0.07)	−0.08 (0.08)
22	−0.30 (0.05)	−0.30 (0.05)	−0.12 (0.06)	−0.31 (0.05)	−0.28 (0.06)
23	−0.20 (0.09)	−0.20 (0.09)	0.04 (0.10)	−0.19 (0.08)	−0.21 (0.09)
24	−0.20 (0.06)	−0.20 (0.06)	−0.01 (0.11)	−0.12 (0.09)	−0.20 (0.07)
25	−0.17 (0.13)	0.17 (0.14)	−0.09 (0.10)	−0.25 (0.10)	−0.13 (0.19)
26	0.12 (0.13)	0.12 (0.13)	0.11 (0.11)	−0.02 (0.11)	0.10 (0.12)
27	−0.32 (0.16)	−0.32 (0.16)	−0.15 (0.16)	−0.29 (0.15)	−0.32 (0.16)
28	−0.32 (0.17)	−0.32 (0.17)	−0.20 (0.15)	−0.51 (0.12)	−0.31 (0.14)
29	−0.08 (0.11)	−0.08 (0.12)	−0.02 (0.14)	−0.09 (0.09)	−0.09 (0.13)
30	−0.32 (0.18)	−0.30 (0.18)	−0.28 (0.19)	−0.33 (0.18)	−0.32 (0.15)
31	−0.10 (0.11)	−0.10 (0.11)	−0.01 (0.09)	−0.19 (0.08)	−0.12 (0.14)
32	−0.10 (0.14)	−0.14 (0.14)	−0.24 (0.10)	−0.22 (0.13)	−0.16 (0.14)
33	0.04 (0.12)	0.04 (0.12)	0 (0.10)	−0.02 (0.09)	0.04 (0.14)
34	−0.02 (0.100)	−0.02 (0.100)	−0.01 (0.10)	−0.06 (0.09)	0.22 (0.11)
35	−0.23 (0.06)	−0.23 (0.06)	−0.04 (0.04)	−0.20 (0.05)	−0.23 (0.07)
36	−0.30 (0.12)	−0.31 (0.13)	0.08 (0.12)	−0.23 (0.16)	−0.27 (0.15)

**Table 6:** Percentage of outliers of bias/relative bias by method CA, method FA, method DMM, method λ_6_, and method LCM in simulation conditions 1–36.

Condition number	Method CA	Method FA	Method DMM	Method λ_6_	Method LCM
1	1.5%	1.3%	1.6%	1.1%	2.9%
2	1.3%	1.7%	1.8%	1.2%	2.0%
3	2.7%	2.4%	2.6%	1.8%	3.2%
4	2.6%	1.8%	2.2%	1.4%	3.3%
5	0.9%	1.0%	1.1%	1.1%	1.2%
6	2.4%	1.9%	1.3%	3.1%	2.7%
7	1.6%	1.2%	1.7%	2.7%	1.4%
8	0.5%	0.6%	0.7%	0.9%	0.4%
9	1.3%	1.4%	1.6%	1.1%	3.2%
10	0.2%	1.7%	0.3%	2.6%	3.1%
11	2.2%	1.8%	0.4%	7.8%	1.6%
12	1.1%	0.7%	0.4%	0.6%	2.2%
13	0.9%	0.7%	1.1%	0.9%	2.2%
14	0.4%	1.2%	0.6%	0.5%	0.9%
15	1.0%	1.3%	0.7%	3.7%	1.4%
16	1.6%	2.0%	1.5%	1.3%	3.7%
17	2.1%	1.7%	1.8%	6.4%	5.7%
18	2.9%	2.3%	1.8%	1.6%	2.7%
19	1.3%	1.4%	0.3%	0.9%	5.5%
20	0.9%	1.4%	1.1%	0.8%	2.0%
21	2.1%	2.9%	1.6%	1.3%	1.5%
22	1.4%	1.1%	1.2%	1.2%	2.3%
23	0.2%	1.2%	1.1%	1.9%	1.7%
24	2.9%	1.7%	1.0%	1.1%	1.7%
25	1.5%	1.4%	2.3%	3.2%	1.8%
26	1.5%	1.7%	1.2%	1.4%	2.1%
27	1.2%	1.5%	2.1%	0.9%	2.2%
28	0.7%	1.3%	0.8%	1.9%	2.7%
29	2.1%	1.6%	0.7%	2.2%	1.4%
30	1.0%	0.3%	0.2%	0.9%	1.4%
31	0.9%	0.8%	1.0%	2.4%	1.7%
32	0.8%	2.4%	0.8%	1.6%	3.2%
33	1.3%	1.1%	0.9%	2.2%	3.1%
34	1.5%	2.7%	1.4%	1.3%	4.1%
35	2.1%	1.6%	0.7%	5.5%	3.6%
36	2.2%	1.7%	1.4%	1.2%	3.1%

**Table 7:** RMSE by method CA, method FA, method DMM, method λ_6_, and method LCM in simulation conditions 1–36.

Condition number	Method CA	Method FA	Method DMM	Method λ_6_	Method LCM
1	0.03	0.03	0.03	0.03	0.04
2	0.04	0.04	0.04	0.04	0.04
3	0.03	0.03	0.03	0.03	0.03
4	0.04	0.04	0.03	0.03	0.04
5	0.03	0.03	0.03	0.03	0.04
6	0.02	0.02	0.02	0.03	0.02
7	0.04	0.04	0.03	0.04	0.05
8	0.03	0.03	0.03	0.02	0.03
9	0.04	0.04	0.04	0.03	0.04
10	0.03	0.03	0.04	0.02	0.04
11	0.03	0.03	0.03	0.03	0.03
12	0.04	0.04	0.04	0.04	0.04
13	0.04	0.04	0.02	0.03	0.04
14	0.04	0.04	0.04	0.04	0.05
15	0.03	0.03	0.02	0.02	0.02
16	0.02	0.02	0.03	0.03	0.04
17	0.03	0.03	0.03	0.03	0.03
18	0.03	0.03	0.03	0.03	0.03
19	0.02	0.02	0.02	0.02	0.02
20	0.03	0.03	0.02	0.02	0.03
21	0.03	0.03	0.02	0.02	0.03
22	0.02	0.02	0.02	0.02	0.03
23	0.03	0.03	0.03	0.03	0.04
24	0.02	0.02	0.02	0.02	0.02
25	0.04	0.04	0.03	0.03	0.03
26	0.02	0.02	0.02	0.02	0.03
27	0.02	0.02	0.02	0.02	0.03
28	0.03	0.03	0.02	0.02	0.03
29	0.03	0.03	0.02	0.03	0.03
30	0.02	0.02	0.03	0.03	0.03
31	0.03	0.03	0.02	0.03	0.03
32	0.03	0.03	0.02	0.03	0.04
33	0.02	0.02	0.02	0.02	0.02
34	0.04	0.04	0.03	0.03	0.05
35	0.02	0.02	0.02	0.02	0.03
36	0.03	0.02	0.02	0.03	0.03

Looking at the percentage of outliers (see Table 6), method λ_6_ produced 6.4% of outliers in simulation condition 17 and 5.5% of outliers in simulation condition 35, and method LCM produced 5.7% of outliers in simulation condition 17. Apart from these exceptions, all other percentages of outliers were less than 5%. Method CA, method FA, method DMM, method λ_6_, and method LCM generated a small percentage of outliers in most simulation conditions.

When considering bias, method LCM produced an IQR of 0.073 in simulation condition 10 and an IQR of 0.090 in simulation condition 7, apart from these two IQRs, other IQRs were not greater than 0.070 (see Table 4). All RMSEs were less than 0.044 (see Table 7). Based on the percentage of outliers of bias, the IQR of bias and the RMSE, method CA, method FA, method DMM, method λ_6_, and method LCM produced acceptable deviation values for estimating the reliability of single-item assessments in most simulation conditions.

Looking at the median of bias, only 13.89% of the medians were positive (see Table 4). Method CA, method FA, method DMM, method λ_6_, and method LCM underestimated the reliability of single-item assessments in most simulation conditions.

### 4.2 Selection of the most precise reliability estimation method

In each simulation condition, we first focused on the percentage of outliers, if the percentage of outliers for a method is greater than 5%, then that method is discarded, as we are not 95% confident that this method can precisely estimate the reliability of single-item assessments. Among the remaining methods for estimating the reliability of single-item assessments, if one method has the smallest absolute value of the median, IQR, RMSE, and the percentage of outliers compared to other methods, then that method is the most precise. If a method has the smallest absolute value of the median while having a comparable deviation (IQR, RMSE, percentage of outliers) to other methods, then this method will be the most precise method for estimating the reliability of single-item assessments. If two methods are indistinguished in the above four dimensions (e.g., two methods are almost identical in the above four dimensions; one method has a smaller absolute value of the median while another has a smaller deviation), we will choose the easier method to estimate the reliability of single-item assessments, e.g., if method CA and method LCM are almost identical in the above four dimensions, we will choose method CA to estimate the reliability of single-item assessments because method CA is much easier to perform than method LCM. Following the above criteria, the most precise method in each simulation condition is shown in Table 8.

**Table 8:** The most precise method for estimating the reliability of single-item assessment in simulation conditions 1–36.

Condition number	The most precise method
1	Method DMM
2	Method DMM
3	Method DMM
4	Method DMM
5	Method DMM
6	Method DMM
7	Method DMM
8	Method LCM
9	Method DMM
10	Method CA
11	Method DMM
12	Method DMM
13	Method DMM
14	Method CA
15	Method DMM
16	Method DMM
17	Method DMM
18	Method DMM
19	Method DMM
20	Method DMM
21	Method DMM
22	Method DMM
23	Method DMM
24	Method DMM
25	Method DMM
26	Method λ_6_
27	Method DMM
28	Method DMM
29	Method DMM
30	Method DMM
31	Method DMM
32	Method CA
33	Method DMM
34	Method DMM
35	Method DMM
36	Method DMM

The conclusions about precision (Table 8) were based on an initial review of the median and IQR of bias, the median and IQR of relative bias, the percentage of outliers of bias, and the RMSE, but in some simulation conditions it was difficult to determine the most precise method based on such an initial review. For example, in simulation condition 30, method FA and method DMM are comparable in terms of deviation (IQR, RMSE, and percentage of outliers), we chose method DMM because the absolute value of the median of bias for method DMM (0.10) was smaller than that for method FA (0.11), while the difference was very small (0.01); could we conclude that method DMM is the most precise method based on such a small difference between two absolute values of the median of bias? To address this issue, we used Kruskal–Wallis tests to examine the difference between group medians in each simulation condition. The Kruskal–Wallis test is a non-parametric equivalent of a one-way ANOVA test, testing whether there is a significant difference between three or more group medians. When a Kruskal–Wallis test shows that there is a significant difference between medians, researchers perform Kruskal–Wallis multiple comparisons to compare the medians of each pair of groups (Rayner and Best, 1997). After running Kruskal–Wallis tests and Kruskal–Wallis multiple comparisons in each simulation condition, the results in Table 8 remained almost the same except for two simulation conditions, condition 14 and condition 30. In simulation condition 14, the Kruskal–Wallis test was significant (*H*(4) = 12.06, *p* < 0.05), but there was no significant difference in the median of bias between method CA and method FA (*p* = 0.62); given that method CA and method FA had comparable deviation, method CA and method FA were both the most precise methods for estimating the reliability of single-item assessments in simulation condition 14. In simulation condition 30, the Kruskal–Wallis test was significant (*H*(4) = 8.73, *p* < 0.05), but there was no significant difference in the median of bias between method FA and method DMM (*p* = 0.17); given that method DMM and method FA were comparable in deviation, method DMM and method FA were both the most precise methods to estimate the reliability of single-item assessments in simulation condition 30. Based on the results in Table 8 and the results of the Kruskal–Wallis test in each simulation condition, the most precise method in each simulation condition is shown in Table 9.

**Table 9:** The most precise method for estimating the reliability of single-item assessment in simulation conditions 1–36 (referenced using Kruskal–Wallis tests).

Condition number	The most precise method
1	Method DMM
2	Method DMM
3	Method DMM
4	Method DMM
5	Method DMM
6	Method DMM
7	Method DMM
8	Method LCM
9	Method DMM
10	Method CA
11	Method DMM
12	Method DMM
13	Method DMM
14	Method CA and method FA
15	Method DMM
16	Method DMM
17	Method DMM
18	Method DMM
19	Method DMM
20	Method DMM
21	Method DMM
22	Method DMM
23	Method DMM
24	Method DMM
25	Method DMM
26	Method λ_6_
27	Method DMM
28	Method DMM
29	Method DMM
30	Method DMM and method FA
31	Method DMM
32	Method CA
33	Method DMM
34	Method DMM
35	Method DMM
36	Method DMM

As shown in Table 9, method DMM is the most precise method for estimating the reliability of single-item assessments in 30 simulation conditions, method LCM is the most precise method in simulation condition 8, method CA is the most precise method in simulation conditions 10 and 32, and method λ_6_ is the most precise method in simulation condition 26. In simulation condition 14, both method CA and method FA are the most precise methods. In simulation condition 30, both method FA and method DMM are the most precise methods.

Out of 36 simulation conditions, method DMM is the most precise method for estimating the reliability of single-item assessments in 31 simulation conditions (in condition 30, both method DMM and method FA are the best methods), method CA is the most precise method in 3 simulation conditions (in condition 14, both method CA and method FA are the most precise). All simulation conditions may occur in real practice, it is a tedious task for researchers to examine the multi-item assessment and the correlation between single-item assessment and multi-item assessment and then decide which method should be adopted. Given that we are 94.44% confident in precisely estimating the reliability of single-item assessments using method DMM and method CA, if researchers want to estimate the reliability of single-item assessment, they should use both method DMM and method CA regardless of test length, discrimination, correlation and sample size; these two reliability estimates should be provided simultaneously to show the range of reliability of single-item assessments.

## 5 Discussion

The present study compared five reliability estimation methods for single-item assessment using a simulation approach. The results imply that reliability estimation by method DMM and method CA should be performed simultaneously to ensure the precision of reliability estimation. However, as shown in Tables 4, 5, out of 36 simulation conditions, only in simulation conditions 15 and 33 did the most precise method, method DMM, produce a median bias of 0 (i.e., method DMM produced an unbiased estimate of the reliability of single-item assessment in these two simulation conditions), in other 34 simulation conditions, even the most precise method did not have a median bias of 0. Simulation conditions 15 and 33 are “ideal” conditions to estimate the reliability of single-item assessments. In simulation conditions 15 and 33, the length of the multi-item assessment is long (18 items), the items in the multi-item assessment are equally discriminating, and the correlation between the single-item assessment and its corresponding multi-item assessment is 0.85. This finding seems to imply that, in order to estimate the reliability of a single-item assessment, researchers should choose a longer multi-item assessment with equally discriminating items that are also expected to correlate highly with the single-item assessment. However, equally discriminating multi-item assessments do not exist for many constructs, and the correlations between the single-item assessment and existing corresponding multi-item assessments may not be as high as 0.85. Ideal conditions, such as simulated in conditions 15 and 33, are usually not achievable in practice. Should researchers spend a lot of time selecting a corresponding multi-item assessment to estimate the reliability of single-item assessment in practice?

We investigated the above question using generalized linear models. In generalized linear models, bias is the dependent variable, simulation conditions (test length, discriminating condition, correlation between single-item assessment and multi-item assessment of the same construct, and sample size), choice of method, and interactions of simulation conditions were chosen as predictors. We transformed these predictors into dummy variables, and chose method CA, short multi-item assessment, equally discriminating multi-item assessment, correlation 0.65, small sample size, correlation 0.65*short multi-item assessment, equally discriminating multi-item assessment*correlation 0.65, and short multi-item assessment*equally discriminating multi-item assessment as reference groups. In model 1, we chose all simulation conditions, method choice, and the interaction of test length and correlation as predictors; in model 2, all simulation conditions, method choice and the interaction of discriminating condition and correlation were predictors; in model 3, all simulation conditions, method choice, the interaction of test length, and discriminating condition were predictors. The results of three generalized linear models are shown in Table 10. In models 1–3, method FA, method LCM, and method λ_6_ produce identical biases as method CA, while method DMM produces significantly smaller biases than method CA, indicating that method DMM is the most precise method for estimating the reliability of single-item assessments; this conclusion is consistent with our previous finding that method DMM is the most precise method in 86% of simulation conditions. In addition, the simulation conditions and interaction predictors in models 1–3 did not affect the bias (these predictors were not significant), which means that researchers do not need to spend a lot of time selecting a corresponding multi-item assessment to estimate the reliability of single-item assessments, researchers can obtain the most precise estimate of the range of reliability of a single-item assessment in about 95% of cases when the length of the multi-item assessment is greater than 6 items, and the correlation between the single-item assessment and the corresponding multi-item assessment is greater than 0.65.

**Table 10:** Coefficient estimates from three generalized linear models.

Predictor	Model 1	Model 2	Model 3
Method FA	0	0	0
Method DMM	−0.05*	−0.07*	−0.05*
Method λ_6_	0	0.01	−0.01
Method LCM	−0.02	0	−0.01
Medium length	0.01	−0.01	0
Long length	−0.01	0.02	0.01
Unequally discriminating	0	0	0
*r* = 0.75	−0.01	0	0.02
*r* = 0.85	0.01	−0.01	0
Large sample size	0	0	0
*r* = 0.65 * medium length	0		
*r* = 0.65 * long length	0		
*r* = 0.75 * short length	−0.01		
*r* = 0.75 * medium length	0.02		
*r* = 0.75 * long length	0		
*r* = 0.85 * short length	0		
*r* = 0.85 * medium length	−0.01		
*r* = 0.85 * long length	0.01		
Equally discriminating * *r* = 0.75		0	
Equally discriminating * *r* = 0.85		−0.01	
Unequally discriminating * *r* = 0.65		0	
Unequally discriminating * *r* = 0.75		0	
Unequally discriminating * *r* = 0.85		0.01	
Equally discriminating * medium length			0
Equally discriminating * long length			0
Unequally discriminating * short length			0.02
Unequally discriminating * medium length			0.01
Unequally discriminating * long length			0

## 6 Conclusion

Methods based on correction for attenuation (method CA), factor analysis (method FA), double monotonicity (method DMM), Guttman’s λ_6_ (method λ_6_), and the latent class model (method LCM) have been developed to estimate the reliability of single-item assessments. In practice, researchers use one or two of these methods to estimate the reliability of single-item assessments, but there has been little research into which method estimates the reliability of single-item assessments most precisely. This study investigated this question using a simulation study. To represent different assessments as comprehensively as possible, this simulation study varied several aspects: test length, the item discrimination parameter, sample size, and the correlation between the single-item assessment and the multi-item assessment of the same construct. The current results suggest that by using both method DMM and method CA simultaneously, researchers can obtain the most precise estimate of the range of reliability of a single-item assessment in about 95% of cases when the length of the multi-item assessment is greater than 6 items, and the correlation between the single-item assessment and the corresponding multi-item assessment is greater than 0.65, while these two methods underestimate the reliability of single-item assessments in the majority of cases.

Single-item assessments have been used in many studies in different areas of research, and reliability estimation is mandatory for any assessment. Because test–retest reliability is sometimes inappropriate for single-item assessments measuring transient constructs, researchers have developed five methods for estimating the reliability of single-item assessments. This study has, for the first time, shown the most precise of these five methods for estimating the reliability of single-item assessments, and has also provided the R code for each method. Our study will encourage and facilitate the use of single-item assessments by psychologists (especially organizational and clinical psychologists).

The multi-item assessments of the same construct in this simulation study were unidimensional, whereas in practice multidimensional multi-item assessments were also used to estimate the reliability of some single-item assessments, since for some constructs, all existing multi-item assessments are multidimensional. In addition, all single-item assessments and corresponding multi-item assessments in this simulation study used a 5-point Likert scale, but in practice, dichotomous single-item assessments were also occasionally used. Further research is needed to investigate the effect of multi-item dimensionality and scale format on the estimation of the reliability of the single-item assessments.

## Data Availability

The original contributions presented in the study are included in the article/Supplementary material, further inquiries can be directed to the corresponding authors.
