# Supplementary material for: Comparison of different reliability estimation methods for single-item assessment: a simulation study
Source: Front Psychol. 2024 Nov 1;15:1482016. doi: 10.3389/fpsyg.2024.1482016 (PMC11568483; doi:10.3389/fpsyg.2024.1482016)
Supplement: Supplementary file 1 [file Data_Sheet_1.DOCX]

**R CODE TO DEDINE THE TRUE RELIABILITY OF SINGLE-ITEM ASSESSMENTS**

# Define the true reliability by simulating a population of N = 1e6.

require(MASS)

set.seed(944507)

design <- list()

sixb1=c(runif(1,-4.2,0),runif(1,-4.2,0),runif(1,-4.2,0),runif(1,-4.2,0),runif(1,-4.2,0),runif(1,-4.2,0), runif(1,-4.2,0))

sixb2=sixb1+c(runif(1,1.4,2.5), runif(1,1.4,2.5),runif(1,1.4,2.5),runif(1,1.4,2.5),runif(1,1.4,2.5),runif(1,1.4,2.5), runif(1,1.4,2.5))

sixb3=sixb2+c(runif(1,1.4,2.5), runif(1,1.4,2.5),runif(1,1.4,2.5),runif(1,1.4,2.5),runif(1,1.4,2.5),runif(1,1.4,2.5), runif(1,1.4,2.5))

sixb4=sixb3+c(runif(1,1.4,2.5), runif(1,1.4,2.5),runif(1,1.4,2.5),runif(1,1.4,2.5),runif(1,1.4,2.5),runif(1,1.4,2.5), runif(1,1.4,2.5))

design[[1]] <- list(Q=2, rho=matrix(c(1,.65,.65,1), ncol = 2), psi=matrix(c(rep(1,6),rep(0,7), runif(1,0.4,2.8)),ncol=2), delta=cbind(sixb1,sixb2,sixb3,sixb4),J=7, N=1e6, m=4)

twelveb1=c(runif(1,-4.2,0),runif(1,-4.2,0),runif(1,-4.2,0),runif(1,-4.2,0),runif(1,-4.2,0),runif(1,-4.2,0), runif(1,-4.2,0), runif(1,-4.2,0), runif(1,-4.2,0), runif(1,-4.2,0), runif(1,-4.2,0), runif(1,-4.2,0), runif(1,-4.2,0))

twelveb2= twelveb1 + c(runif(1,1.4,2.5), runif(1,1.4,2.5), runif(1,1.4,2.5), runif(1,1.4,2.5), runif(1,1.4,2.5),runif(1,1.4,2.5), runif(1,1.4,2.5),runif(1,1.4,2.5),runif(1,1.4,2.5),runif(1,1.4,2.5),runif(1,1.4,2.5),runif(1,1.4,2.5), runif(1,1.4,2.5))

twelveb3=twelveb2+ c(runif(1,1.4,2.5), runif(1,1.4,2.5), runif(1,1.4,2.5), runif(1,1.4,2.5), runif(1,1.4,2.5),runif(1,1.4,2.5), runif(1,1.4,2.5),runif(1,1.4,2.5),runif(1,1.4,2.5),runif(1,1.4,2.5),runif(1,1.4,2.5),runif(1,1.4,2.5), runif(1,1.4,2.5))

twelveb4=twelveb3+ c(runif(1,1.4,2.5), runif(1,1.4,2.5), runif(1,1.4,2.5), runif(1,1.4,2.5), runif(1,1.4,2.5),runif(1,1.4,2.5), runif(1,1.4,2.5),runif(1,1.4,2.5),runif(1,1.4,2.5),runif(1,1.4,2.5),runif(1,1.4,2.5),runif(1,1.4,2.5), runif(1,1.4,2.5))

design[[2]] <- list(Q=2, rho=matrix(c(1,.65,.65,1), ncol = 2), psi=matrix(c(rep(1,12),rep(0,13), runif(1,0.4,2.8)),ncol=2), delta=cbind(twelveb1,twelveb2,twelveb3,twelveb4),J=13, N=1e6, m=4)

eighteenb1=c(runif(1,-4.2,0),runif(1,-4.2,0),runif(1,-4.2,0),runif(1,-4.2,0),runif(1,-4.2,0),runif(1,-4.2,0), runif(1,-4.2,0), runif(1,-4.2,0), runif(1,-4.2,0), runif(1,-4.2,0), runif(1,-4.2,0), runif(1,-4.2,0), runif(1,-4.2,0), runif(1,-4.2,0), runif(1,-4.2,0), runif(1,-4.2,0), runif(1,-4.2,0), runif(1,-4.2,0), runif(1,-4.2,0))

eighteenb2= eighteenb1+ c(runif(1,1.4,2.5), runif(1,1.4,2.5), runif(1,1.4,2.5), runif(1,1.4,2.5), runif(1,1.4,2.5), runif(1,1.4,2.5), runif(1,1.4,2.5), runif(1,1.4,2.5),runif(1,1.4,2.5), runif(1,1.4,2.5), runif(1,1.4,2.5),runif(1,1.4,2.5), runif(1,1.4,2.5),runif(1,1.4,2.5),runif(1,1.4,2.5),runif(1,1.4,2.5),runif(1,1.4,2.5),runif(1,1.4,2.5), runif(1,1.4,2.5))

eighteenb3= eighteenb2+ c(runif(1,1.4,2.5), runif(1,1.4,2.5), runif(1,1.4,2.5), runif(1,1.4,2.5), runif(1,1.4,2.5), runif(1,1.4,2.5), runif(1,1.4,2.5), runif(1,1.4,2.5),runif(1,1.4,2.5), runif(1,1.4,2.5), runif(1,1.4,2.5),runif(1,1.4,2.5), runif(1,1.4,2.5),runif(1,1.4,2.5),runif(1,1.4,2.5),runif(1,1.4,2.5),runif(1,1.4,2.5),runif(1,1.4,2.5), runif(1,1.4,2.5))

eighteenb4= eighteenb3+ c(runif(1,1.4,2.5), runif(1,1.4,2.5), runif(1,1.4,2.5), runif(1,1.4,2.5), runif(1,1.4,2.5), runif(1,1.4,2.5), runif(1,1.4,2.5), runif(1,1.4,2.5),runif(1,1.4,2.5), runif(1,1.4,2.5), runif(1,1.4,2.5),runif(1,1.4,2.5), runif(1,1.4,2.5),runif(1,1.4,2.5),runif(1,1.4,2.5),runif(1,1.4,2.5),runif(1,1.4,2.5),runif(1,1.4,2.5), runif(1,1.4,2.5))

design[[3]] <- list(Q=2, rho=matrix(c(1,.65,.65,1), ncol = 2), psi=matrix(c(rep(1,18),rep(0,19), runif(1,0.4,2.8)),ncol=2), delta=cbind(eighteenb1, eighteenb2, eighteenb3, eighteenb4),J=19, N=1e6, m=4)

design[[4]] <- list(Q=2, rho=matrix(c(1,.65,.65,1), ncol = 2), psi=matrix(c(runif(1,0.4,2.8), runif(1,0.4,2.8), runif(1,0.4,2.8), runif(1,0.4,2.8), runif(1,0.4,2.8), runif(1,0.4,2.8), rep(0,7), runif(1,0.4,2.8)),ncol=2),

delta=cbind(sixb1,sixb2,sixb3,sixb4),J=7, N=1e6, m=4)

design[[5]] <- list(Q=2, rho=matrix(c(1,.65,.65,1), ncol = 2), psi=matrix(c(runif(1,0.4,2.8), runif(1,0.4,2.8), runif(1,0.4,2.8), runif(1,0.4,2.8), runif(1,0.4,2.8), runif(1,0.4,2.8), runif(1,0.4,2.8), runif(1,0.4,2.8), runif(1,0.4,2.8), runif(1,0.4,2.8), runif(1,0.4,2.8), runif(1,0.4,2.8), rep(0,13), runif(1,0.4,2.8)),ncol=2), delta=cbind(twelveb1,twelveb2,twelveb3,twelveb4),J=13, N=1e6, m=4)

design[[6]] <- list(Q=2, rho=matrix(c(1,.65,.65,1), ncol = 2), psi=matrix(c(runif(1,0.4,2.8), runif(1,0.4,2.8), runif(1,0.4,2.8), runif(1,0.4,2.8), runif(1,0.4,2.8), runif(1,0.4,2.8), runif(1,0.4,2.8), runif(1,0.4,2.8), runif(1,0.4,2.8), runif(1,0.4,2.8), runif(1,0.4,2.8), runif(1,0.4,2.8), runif(1,0.4,2.8), runif(1,0.4,2.8), runif(1,0.4,2.8), runif(1,0.4,2.8), runif(1,0.4,2.8), runif(1,0.4,2.8),rep(0,19), runif(1,0.4,2.8)),ncol=2), delta=cbind(eighteenb1, eighteenb2, eighteenb3, eighteenb4),J=19, N=1e6, m=4)

design[[7]] <- list(Q=2, rho=matrix(c(1,.75,.75,1), ncol = 2), psi=matrix(c(rep(1,6),rep(0,7), runif(1,0.4,2.8)),ncol=2), delta=cbind(sixb1,sixb2,sixb3,sixb4),J=7, N=1e6, m=4)

design[[8]] <- list(Q=2, rho=matrix(c(1,.75,.75,1), ncol = 2), psi=matrix(c(rep(1,12),rep(0,13), runif(1,0.4,2.8)),ncol=2), delta=cbind(twelveb1,twelveb2,twelveb3,twelveb4),J=13, N=1e6, m=4)

design[[9]] <- list(Q=2, rho=matrix(c(1,.75,.75,1), ncol = 2), psi=matrix(c(rep(1,18),rep(0,19), runif(1,0.4,2.8)),ncol=2), delta=cbind(eighteenb1, eighteenb2, eighteenb3, eighteenb4),J=19, N=1e6, m=4)

design[[10]] <- list(Q=2, rho=matrix(c(1,.75,.75,1), ncol = 2), psi=matrix(c(runif(1,0.4,2.8), runif(1,0.4,2.8), runif(1,0.4,2.8), runif(1,0.4,2.8), runif(1,0.4,2.8), runif(1,0.4,2.8), rep(0,7), runif(1,0.4,2.8)),ncol=2),

delta=cbind(sixb1,sixb2,sixb3,sixb4),J=7, N=1e6, m=4)

design[[11]] <- list(Q=2, rho=matrix(c(1,.75,.75,1), ncol = 2), psi=matrix(c(runif(1,0.4,2.8), runif(1,0.4,2.8), runif(1,0.4,2.8), runif(1,0.4,2.8), runif(1,0.4,2.8), runif(1,0.4,2.8), runif(1,0.4,2.8), runif(1,0.4,2.8), runif(1,0.4,2.8), runif(1,0.4,2.8), runif(1,0.4,2.8), runif(1,0.4,2.8), rep(0,13), runif(1,0.4,2.8)),ncol=2), delta=cbind(twelveb1,twelveb2,twelveb3,twelveb4),J=13, N=1e6, m=4)

design[[12]] <- list(Q=2, rho=matrix(c(1,.75,.75,1), ncol = 2), psi=matrix(c(runif(1,0.4,2.8), runif(1,0.4,2.8), runif(1,0.4,2.8), runif(1,0.4,2.8), runif(1,0.4,2.8), runif(1,0.4,2.8), runif(1,0.4,2.8), runif(1,0.4,2.8), runif(1,0.4,2.8), runif(1,0.4,2.8), runif(1,0.4,2.8), runif(1,0.4,2.8), runif(1,0.4,2.8), runif(1,0.4,2.8), runif(1,0.4,2.8), runif(1,0.4,2.8), runif(1,0.4,2.8), runif(1,0.4,2.8),rep(0,19), runif(1,0.4,2.8)),ncol=2), delta=cbind(eighteenb1, eighteenb2, eighteenb3, eighteenb4),J=19, N=1e6, m=4)

design[[13]] <- list(Q=2, rho=matrix(c(1,.85,.85,1), ncol = 2), psi=matrix(c(rep(1,6),rep(0,7), runif(1,0.4,2.8)),ncol=2), delta=cbind(sixb1,sixb2,sixb3,sixb4),J=7, N=1e6, m=4)

design[[14]] <- list(Q=2, rho=matrix(c(1,.85,.85,1), ncol = 2), psi=matrix(c(rep(1,12),rep(0,13), runif(1,0.4,2.8)),ncol=2), delta=cbind(twelveb1,twelveb2,twelveb3,twelveb4),J=13, N=1e6, m=4)

design[[15]] <- list(Q=2, rho=matrix(c(1,.85,.85,1), ncol = 2), psi=matrix(c(rep(1,18),rep(0,19), runif(1,0.4,2.8)),ncol=2), delta=cbind(eighteenb1, eighteenb2, eighteenb3, eighteenb4),J=19, N=1e6, m=4)

design[[16]] <- list(Q=2, rho=matrix(c(1,.85,.85,1), ncol = 2), psi=matrix(c(runif(1,0.4,2.8), runif(1,0.4,2.8), runif(1,0.4,2.8), runif(1,0.4,2.8), runif(1,0.4,2.8), runif(1,0.4,2.8), rep(0,7), runif(1,0.4,2.8)),ncol=2),

delta=cbind(sixb1,sixb2,sixb3,sixb4),J=7, N=1e6, m=4)

design[[17]] <- list(Q=2, rho=matrix(c(1,.85,.85,1), ncol = 2), psi=matrix(c(runif(1,0.4,2.8), runif(1,0.4,2.8), runif(1,0.4,2.8), runif(1,0.4,2.8), runif(1,0.4,2.8), runif(1,0.4,2.8), runif(1,0.4,2.8), runif(1,0.4,2.8), runif(1,0.4,2.8), runif(1,0.4,2.8), runif(1,0.4,2.8), runif(1,0.4,2.8), rep(0,13), runif(1,0.4,2.8)),ncol=2), delta=cbind(twelveb1,twelveb2,twelveb3,twelveb4),J=13, N=1e6, m=4)

design[[18]] <- list(Q=2, rho=matrix(c(1,.85,.85,1), ncol = 2), psi=matrix(c(runif(1,0.4,2.8), runif(1,0.4,2.8), runif(1,0.4,2.8), runif(1,0.4,2.8), runif(1,0.4,2.8), runif(1,0.4,2.8), runif(1,0.4,2.8), runif(1,0.4,2.8), runif(1,0.4,2.8), runif(1,0.4,2.8), runif(1,0.4,2.8), runif(1,0.4,2.8), runif(1,0.4,2.8), runif(1,0.4,2.8), runif(1,0.4,2.8), runif(1,0.4,2.8), runif(1,0.4,2.8), runif(1,0.4,2.8),rep(0,19), runif(1,0.4,2.8)),ncol=2), delta=cbind(eighteenb1, eighteenb2, eighteenb3, eighteenb4),J=19, N=1e6, m=4)

rho.XX <- list()

for(i in 1:18){

# parameters

Q <- design[[i]]$Q

rho <- design[[i]]$rho

N <- design[[i]]$N

J <- design[[i]]$J

m <- design[[i]]$m

theta <- matrix(mvrnorm(N,matrix(0,Q,1),rho),ncol = Q) # NxQ

psi <- design[[i]]$psi # JxQ

delta <- design[[i]]$delta # Jxm

# P(Xj >= x|theta)

cum.prob <- list()

for (j in 1:J){

o <- 0

for (q in 1:Q){

o <- o + psi[j,q] * (matrix(rep(theta[,q], m),N,m) - matrix(rep(delta[j,], each = N),N,m))

}

cum.prob[[j]] <- exp(o)/(1+exp(o))

}

# generate data

data <- matrix(NA,N,J)

for (j in 1:J) data[,j] <- apply((sign(cum.prob[[j]] - matrix(runif(N*m),N,m)) + 1)/2,1,sum)

# rho_XX'

EX.theta <- lapply(cum.prob, function(x) matrix(apply(x,1,sum),N,1))

true.score <- matrix(unlist(EX.theta),N,J)

test.score <- data

rho.XX[[i]] <- matrix(NA, nrow = J, ncol = 1)

for (j in 1:J) rho.XX[[i]][j] <- var(true.score[,j])/var(test.score[,j])

}

**R CODE TO GENERATE DATA UNDER EACH SIMULATION CONDITOON**

# Define the simulation conditions

design <- list()

data.files <- list()

drive <- "C:/scholar/graduate school/dissertation/data"

rsim <- 1000 # In each simulation condition, 1000 data sets are drawn

design[[1]] <- list(Q=2, rho=matrix(c(1,.65,.65,1), ncol = 2), psi=matrix(c(rep(1,6),rep(0,7), runif(1,0.4,2.8)),ncol=2), delta=cbind(sixb1,sixb2,sixb3,sixb4),J=7, N=400, m=4)

design[[2]] <- list(Q=2, rho=matrix(c(1,.65,.65,1), ncol = 2), psi=matrix(c(rep(1,12),rep(0,13), runif(1,0.4,2.8)),ncol=2), delta=cbind(twelveb1,twelveb2,twelveb3,twelveb4),J=13, N=400, m=4)

design[[3]] <- list(Q=2, rho=matrix(c(1,.65,.65,1), ncol = 2), psi=matrix(c(rep(1,18),rep(0,19), runif(1,0.4,2.8)),ncol=2), delta=cbind(eighteenb1, eighteenb2, eighteenb3, eighteenb4),J=19, N=400, m=4)

design[[4]] <- list(Q=2, rho=matrix(c(1,.65,.65,1), ncol = 2), psi=matrix(c(runif(1,0.4,2.8), runif(1,0.4,2.8), runif(1,0.4,2.8), runif(1,0.4,2.8), runif(1,0.4,2.8), runif(1,0.4,2.8), rep(0,7), runif(1,0.4,2.8)),ncol=2),

delta=cbind(sixb1,sixb2,sixb3,sixb4),J=7, N=400, m=4)

design[[5]] <- list(Q=2, rho=matrix(c(1,.65,.65,1), ncol = 2), psi=matrix(c(runif(1,0.4,2.8), runif(1,0.4,2.8), runif(1,0.4,2.8), runif(1,0.4,2.8), runif(1,0.4,2.8), runif(1,0.4,2.8), runif(1,0.4,2.8), runif(1,0.4,2.8), runif(1,0.4,2.8), runif(1,0.4,2.8), runif(1,0.4,2.8), runif(1,0.4,2.8), rep(0,13), runif(1,0.4,2.8)),ncol=2), delta=cbind(twelveb1,twelveb2,twelveb3,twelveb4),J=13, N=400, m=4)

design[[6]] <- list(Q=2, rho=matrix(c(1,.65,.65,1), ncol = 2), psi=matrix(c(runif(1,0.4,2.8), runif(1,0.4,2.8), runif(1,0.4,2.8), runif(1,0.4,2.8), runif(1,0.4,2.8), runif(1,0.4,2.8), runif(1,0.4,2.8), runif(1,0.4,2.8), runif(1,0.4,2.8), runif(1,0.4,2.8), runif(1,0.4,2.8), runif(1,0.4,2.8), runif(1,0.4,2.8), runif(1,0.4,2.8), runif(1,0.4,2.8), runif(1,0.4,2.8), runif(1,0.4,2.8), runif(1,0.4,2.8),rep(0,19), runif(1,0.4,2.8)),ncol=2), delta=cbind(eighteenb1, eighteenb2, eighteenb3, eighteenb4),J=19, N=400, m=4)

design[[7]] <- list(Q=2, rho=matrix(c(1,.75,.75,1), ncol = 2), psi=matrix(c(rep(1,6),rep(0,7), runif(1,0.4,2.8)),ncol=2), delta=cbind(sixb1,sixb2,sixb3,sixb4),J=7, N=400, m=4)

design[[8]] <- list(Q=2, rho=matrix(c(1,.75,.75,1), ncol = 2), psi=matrix(c(rep(1,12),rep(0,13), runif(1,0.4,2.8)),ncol=2), delta=cbind(twelveb1,twelveb2,twelveb3,twelveb4),J=13, N=400, m=4)

design[[9]] <- list(Q=2, rho=matrix(c(1,.75,.75,1), ncol = 2), psi=matrix(c(rep(1,18),rep(0,19), runif(1,0.4,2.8)),ncol=2), delta=cbind(eighteenb1, eighteenb2, eighteenb3, eighteenb4),J=19, N=400, m=4)

design[[10]] <- list(Q=2, rho=matrix(c(1,.75,.75,1), ncol = 2), psi=matrix(c(runif(1,0.4,2.8), runif(1,0.4,2.8), runif(1,0.4,2.8), runif(1,0.4,2.8), runif(1,0.4,2.8), runif(1,0.4,2.8), rep(0,7), runif(1,0.4,2.8)),ncol=2),

delta=cbind(sixb1,sixb2,sixb3,sixb4),J=7, N=400, m=4)

design[[11]] <- list(Q=2, rho=matrix(c(1,.75,.75,1), ncol = 2), psi=matrix(c(runif(1,0.4,2.8), runif(1,0.4,2.8), runif(1,0.4,2.8), runif(1,0.4,2.8), runif(1,0.4,2.8), runif(1,0.4,2.8), runif(1,0.4,2.8), runif(1,0.4,2.8), runif(1,0.4,2.8), runif(1,0.4,2.8), runif(1,0.4,2.8), runif(1,0.4,2.8), rep(0,13), runif(1,0.4,2.8)),ncol=2), delta=cbind(twelveb1,twelveb2,twelveb3,twelveb4),J=13, N=400, m=4)

design[[12]] <- list(Q=2, rho=matrix(c(1,.75,.75,1), ncol = 2), psi=matrix(c(runif(1,0.4,2.8), runif(1,0.4,2.8), runif(1,0.4,2.8), runif(1,0.4,2.8), runif(1,0.4,2.8), runif(1,0.4,2.8), runif(1,0.4,2.8), runif(1,0.4,2.8), runif(1,0.4,2.8), runif(1,0.4,2.8), runif(1,0.4,2.8), runif(1,0.4,2.8), runif(1,0.4,2.8), runif(1,0.4,2.8), runif(1,0.4,2.8), runif(1,0.4,2.8), runif(1,0.4,2.8), runif(1,0.4,2.8),rep(0,19), runif(1,0.4,2.8)),ncol=2), delta=cbind(eighteenb1, eighteenb2, eighteenb3, eighteenb4),J=19, N=400, m=4)

design[[13]] <- list(Q=2, rho=matrix(c(1,.85,.85,1), ncol = 2), psi=matrix(c(rep(1,6),rep(0,7), runif(1,0.4,2.8)),ncol=2), delta=cbind(sixb1,sixb2,sixb3,sixb4),J=7, N=400, m=4)

design[[14]] <- list(Q=2, rho=matrix(c(1,.85,.85,1), ncol = 2), psi=matrix(c(rep(1,12),rep(0,13), runif(1,0.4,2.8)),ncol=2), delta=cbind(twelveb1,twelveb2,twelveb3,twelveb4),J=13, N=400, m=4)

design[[15]] <- list(Q=2, rho=matrix(c(1,.85,.85,1), ncol = 2), psi=matrix(c(rep(1,18),rep(0,19), runif(1,0.4,2.8)),ncol=2), delta=cbind(eighteenb1, eighteenb2, eighteenb3, eighteenb4),J=19, N=400, m=4)

design[[16]] <- list(Q=2, rho=matrix(c(1,.85,.85,1), ncol = 2), psi=matrix(c(runif(1,0.4,2.8), runif(1,0.4,2.8), runif(1,0.4,2.8), runif(1,0.4,2.8), runif(1,0.4,2.8), runif(1,0.4,2.8), rep(0,7), runif(1,0.4,2.8)),ncol=2),

delta=cbind(sixb1,sixb2,sixb3,sixb4),J=7, N=400, m=4)

design[[17]] <- list(Q=2, rho=matrix(c(1,.85,.85,1), ncol = 2), psi=matrix(c(runif(1,0.4,2.8), runif(1,0.4,2.8), runif(1,0.4,2.8), runif(1,0.4,2.8), runif(1,0.4,2.8), runif(1,0.4,2.8), runif(1,0.4,2.8), runif(1,0.4,2.8), runif(1,0.4,2.8), runif(1,0.4,2.8), runif(1,0.4,2.8), runif(1,0.4,2.8), rep(0,13), runif(1,0.4,2.8)),ncol=2), delta=cbind(twelveb1,twelveb2,twelveb3,twelveb4),J=13, N=400, m=4)

design[[18]] <- list(Q=2, rho=matrix(c(1,.85,.85,1), ncol = 2), psi=matrix(c(runif(1,0.4,2.8), runif(1,0.4,2.8), runif(1,0.4,2.8), runif(1,0.4,2.8), runif(1,0.4,2.8), runif(1,0.4,2.8), runif(1,0.4,2.8), runif(1,0.4,2.8), runif(1,0.4,2.8), runif(1,0.4,2.8), runif(1,0.4,2.8), runif(1,0.4,2.8), runif(1,0.4,2.8), runif(1,0.4,2.8), runif(1,0.4,2.8), runif(1,0.4,2.8), runif(1,0.4,2.8), runif(1,0.4,2.8),rep(0,19), runif(1,0.4,2.8)),ncol=2), delta=cbind(eighteenb1, eighteenb2, eighteenb3, eighteenb4),J=19, N=400, m=4)

design[[19]] <- list(Q=2, rho=matrix(c(1,.65,.65,1), ncol = 2), psi=matrix(c(rep(1,6),rep(0,7), runif(1,0.4,2.8)),ncol=2), delta=cbind(sixb1,sixb2,sixb3,sixb4),J=7, N=1000, m=4)

design[[20]] <- list(Q=2, rho=matrix(c(1,.65,.65,1), ncol = 2), psi=matrix(c(rep(1,12),rep(0,13), runif(1,0.4,2.8)),ncol=2), delta=cbind(twelveb1,twelveb2,twelveb3,twelveb4),J=13, N=1000, m=4)

design[[21]] <- list(Q=2, rho=matrix(c(1,.65,.65,1), ncol = 2), psi=matrix(c(rep(1,18),rep(0,19), runif(1,0.4,2.8)),ncol=2), delta=cbind(eighteenb1, eighteenb2, eighteenb3, eighteenb4),J=19, N=1000, m=4)

design[[22]] <- list(Q=2, rho=matrix(c(1,.65,.65,1), ncol = 2), psi=matrix(c(runif(1,0.4,2.8), runif(1,0.4,2.8), runif(1,0.4,2.8), runif(1,0.4,2.8), runif(1,0.4,2.8), runif(1,0.4,2.8), rep(0,7), runif(1,0.4,2.8)),ncol=2),

delta=cbind(sixb1,sixb2,sixb3,sixb4),J=7, N=1000, m=4)

design[[23]] <- list(Q=2, rho=matrix(c(1,.65,.65,1), ncol = 2), psi=matrix(c(runif(1,0.4,2.8), runif(1,0.4,2.8), runif(1,0.4,2.8), runif(1,0.4,2.8), runif(1,0.4,2.8), runif(1,0.4,2.8), runif(1,0.4,2.8), runif(1,0.4,2.8), runif(1,0.4,2.8), runif(1,0.4,2.8), runif(1,0.4,2.8), runif(1,0.4,2.8), rep(0,13), runif(1,0.4,2.8)),ncol=2), delta=cbind(twelveb1,twelveb2,twelveb3,twelveb4),J=13, N=1000, m=4)

design[[24]] <- list(Q=2, rho=matrix(c(1,.65,.65,1), ncol = 2), psi=matrix(c(runif(1,0.4,2.8), runif(1,0.4,2.8), runif(1,0.4,2.8), runif(1,0.4,2.8), runif(1,0.4,2.8), runif(1,0.4,2.8), runif(1,0.4,2.8), runif(1,0.4,2.8), runif(1,0.4,2.8), runif(1,0.4,2.8), runif(1,0.4,2.8), runif(1,0.4,2.8), runif(1,0.4,2.8), runif(1,0.4,2.8), runif(1,0.4,2.8), runif(1,0.4,2.8), runif(1,0.4,2.8), runif(1,0.4,2.8),rep(0,19), runif(1,0.4,2.8)),ncol=2), delta=cbind(eighteenb1, eighteenb2, eighteenb3, eighteenb4),J=19, N=1000, m=4)

design[[25]] <- list(Q=2, rho=matrix(c(1,.75,.75,1), ncol = 2), psi=matrix(c(rep(1,6),rep(0,7), runif(1,0.4,2.8)),ncol=2), delta=cbind(sixb1,sixb2,sixb3,sixb4),J=7, N=1000, m=4)

design[[26]] <- list(Q=2, rho=matrix(c(1,.75,.75,1), ncol = 2), psi=matrix(c(rep(1,12),rep(0,13), runif(1,0.4,2.8)),ncol=2), delta=cbind(twelveb1,twelveb2,twelveb3,twelveb4),J=13, N=1000, m=4)

design[[27]] <- list(Q=2, rho=matrix(c(1,.75,.75,1), ncol = 2), psi=matrix(c(rep(1,18),rep(0,19), runif(1,0.4,2.8)),ncol=2), delta=cbind(eighteenb1, eighteenb2, eighteenb3, eighteenb4),J=19, N=1000, m=4)

design[[28]] <- list(Q=2, rho=matrix(c(1,.75,.75,1), ncol = 2), psi=matrix(c(runif(1,0.4,2.8), runif(1,0.4,2.8), runif(1,0.4,2.8), runif(1,0.4,2.8), runif(1,0.4,2.8), runif(1,0.4,2.8), rep(0,7), runif(1,0.4,2.8)),ncol=2),

delta=cbind(sixb1,sixb2,sixb3,sixb4),J=7, N=1000, m=4)

design[[29]] <- list(Q=2, rho=matrix(c(1,.75,.75,1), ncol = 2), psi=matrix(c(runif(1,0.4,2.8), runif(1,0.4,2.8), runif(1,0.4,2.8), runif(1,0.4,2.8), runif(1,0.4,2.8), runif(1,0.4,2.8), runif(1,0.4,2.8), runif(1,0.4,2.8), runif(1,0.4,2.8), runif(1,0.4,2.8), runif(1,0.4,2.8), runif(1,0.4,2.8), rep(0,13), runif(1,0.4,2.8)),ncol=2), delta=cbind(twelveb1,twelveb2,twelveb3,twelveb4),J=13, N=1000, m=4)

design[[30]] <- list(Q=2, rho=matrix(c(1,.75,.75,1), ncol = 2), psi=matrix(c(runif(1,0.4,2.8), runif(1,0.4,2.8), runif(1,0.4,2.8), runif(1,0.4,2.8), runif(1,0.4,2.8), runif(1,0.4,2.8), runif(1,0.4,2.8), runif(1,0.4,2.8), runif(1,0.4,2.8), runif(1,0.4,2.8), runif(1,0.4,2.8), runif(1,0.4,2.8), runif(1,0.4,2.8), runif(1,0.4,2.8), runif(1,0.4,2.8), runif(1,0.4,2.8), runif(1,0.4,2.8), runif(1,0.4,2.8),rep(0,19), runif(1,0.4,2.8)),ncol=2), delta=cbind(eighteenb1, eighteenb2, eighteenb3, eighteenb4),J=19, N=1000, m=4)

design[[31]] <- list(Q=2, rho=matrix(c(1,.85,.85,1), ncol = 2), psi=matrix(c(rep(1,6),rep(0,7), runif(1,0.4,2.8)),ncol=2), delta=cbind(sixb1,sixb2,sixb3,sixb4),J=7, N=1000, m=4)

design[[32]] <- list(Q=2, rho=matrix(c(1,.85,.85,1), ncol = 2), psi=matrix(c(rep(1,12),rep(0,13), runif(1,0.4,2.8)),ncol=2), delta=cbind(twelveb1,twelveb2,twelveb3,twelveb4),J=13, N=1000, m=4)

design[[33]] <- list(Q=2, rho=matrix(c(1,.85,.85,1), ncol = 2), psi=matrix(c(rep(1,18),rep(0,19), runif(1,0.4,2.8)),ncol=2), delta=cbind(eighteenb1, eighteenb2, eighteenb3, eighteenb4),J=19, N=1000, m=4)

design[[34]] <- list(Q=2, rho=matrix(c(1,.85,.85,1), ncol = 2), psi=matrix(c(runif(1,0.4,2.8), runif(1,0.4,2.8), runif(1,0.4,2.8), runif(1,0.4,2.8), runif(1,0.4,2.8), runif(1,0.4,2.8), rep(0,7), runif(1,0.4,2.8)),ncol=2),

delta=cbind(sixb1,sixb2,sixb3,sixb4),J=7, N=1000, m=4)

design[[35]] <- list(Q=2, rho=matrix(c(1,.85,.85,1), ncol = 2), psi=matrix(c(runif(1,0.4,2.8), runif(1,0.4,2.8), runif(1,0.4,2.8), runif(1,0.4,2.8), runif(1,0.4,2.8), runif(1,0.4,2.8), runif(1,0.4,2.8), runif(1,0.4,2.8), runif(1,0.4,2.8), runif(1,0.4,2.8), runif(1,0.4,2.8), runif(1,0.4,2.8), rep(0,13), runif(1,0.4,2.8)),ncol=2), delta=cbind(twelveb1,twelveb2,twelveb3,twelveb4),J=13, N=1000, m=4)

design[[36]] <- list(Q=2, rho=matrix(c(1,.85,.85,1), ncol = 2), psi=matrix(c(runif(1,0.4,2.8), runif(1,0.4,2.8), runif(1,0.4,2.8), runif(1,0.4,2.8), runif(1,0.4,2.8), runif(1,0.4,2.8), runif(1,0.4,2.8), runif(1,0.4,2.8), runif(1,0.4,2.8), runif(1,0.4,2.8), runif(1,0.4,2.8), runif(1,0.4,2.8), runif(1,0.4,2.8), runif(1,0.4,2.8), runif(1,0.4,2.8), runif(1,0.4,2.8), runif(1,0.4,2.8), runif(1,0.4,2.8),rep(0,19), runif(1,0.4,2.8)),ncol=2), delta=cbind(eighteenb1, eighteenb2, eighteenb3, eighteenb4),J=19, N=1000, m=4)

set.seed(3366499)

# Loop to generate data according to the defined parameters

for (i in 1:36){

Q <- design[[i]]$Q

rho <- design[[i]]$rho

psi <- design[[i]]$psi

delta <- design[[i]]$delta

J <- design[[i]]$J

N <- design[[i]]$N

m <- design[[i]]$m

data.files[[i]] <- list()

for (r in 1:rsim) {

data.name <- paste("DES", i, "DATA", substr(as.character(10000+r),2,5), ".dat", sep = "")

data.files[[i]][[r]] <- paste(i,r, sep=",") #paste0(drive,data.name)

print(data.name,fill=T)

theta <- matrix(mvrnorm(N,matrix(0,Q,1),rho),ncol=Q) # draw the thetas cum.prob <- list() # to store the cumulative probabilities

for (j in 1:J){

Z <- 0

for (q in 1:Q) {

Z <- Z + psi[j,q] * (matrix(rep(theta[,q], m), N, m) - matrix(rep(delta[j,], each = N), N, m))

}

cum.prob[[j]] <- exp(Z)/(1+exp(Z))

}

# generate data

data <- matrix(NA,N,J)

for (j in 1:J) data[,j] <- apply((sign(cum.prob[[j]]-matrix(runif(N*m),N,m)) +1)/2,1,sum)

write(t(data)+1,file=paste0(drive,data.name),ncolumns=J)

}

}

**R CODE TO ESTIMATE THE RELIABILITY OF SINGLE-ITEM ASSESSMENT UNDER EACH SIMULATION CONDITION WITH METHOD CA, METHOD FA, NETHOD DMM, METHOD λ_6_, AND METHOD LCM**

install.packages("poLCA", dependencies=TRUE)

install.packages("scatterplot3d", dependencies=TRUE)

install.packages("psych", dependencies=TRUE)

library(psych)

require(MASS)

sixb1=c(runif(1,-4.2,0),runif(1,-4.2,0),runif(1,-4.2,0),runif(1,-4.2,0),runif(1,-4.2,0),runif(1,-4.2,0), runif(1,-4.2,0))

sixb2=sixb1+c(runif(1,1.4,2.5), runif(1,1.4,2.5),runif(1,1.4,2.5),runif(1,1.4,2.5),runif(1,1.4,2.5),runif(1,1.4,2.5), runif(1,1.4,2.5))

sixb3=sixb2+c(runif(1,1.4,2.5), runif(1,1.4,2.5),runif(1,1.4,2.5),runif(1,1.4,2.5),runif(1,1.4,2.5),runif(1,1.4,2.5), runif(1,1.4,2.5))

sixb4=sixb3+c(runif(1,1.4,2.5), runif(1,1.4,2.5),runif(1,1.4,2.5),runif(1,1.4,2.5),runif(1,1.4,2.5),runif(1,1.4,2.5), runif(1,1.4,2.5))

twelveb1=c(runif(1,-4.2,0),runif(1,-4.2,0),runif(1,-4.2,0),runif(1,-4.2,0),runif(1,-4.2,0),runif(1,-4.2,0), runif(1,-4.2,0), runif(1,-4.2,0), runif(1,-4.2,0), runif(1,-4.2,0), runif(1,-4.2,0), runif(1,-4.2,0), runif(1,-4.2,0))

twelveb2= twelveb1 + c(runif(1,1.4,2.5), runif(1,1.4,2.5), runif(1,1.4,2.5), runif(1,1.4,2.5), runif(1,1.4,2.5),runif(1,1.4,2.5), runif(1,1.4,2.5),runif(1,1.4,2.5),runif(1,1.4,2.5),runif(1,1.4,2.5),runif(1,1.4,2.5),runif(1,1.4,2.5), runif(1,1.4,2.5))

twelveb3=twelveb2+ c(runif(1,1.4,2.5), runif(1,1.4,2.5), runif(1,1.4,2.5), runif(1,1.4,2.5), runif(1,1.4,2.5),runif(1,1.4,2.5), runif(1,1.4,2.5),runif(1,1.4,2.5),runif(1,1.4,2.5),runif(1,1.4,2.5),runif(1,1.4,2.5),runif(1,1.4,2.5), runif(1,1.4,2.5))

twelveb4=twelveb3+ c(runif(1,1.4,2.5), runif(1,1.4,2.5), runif(1,1.4,2.5), runif(1,1.4,2.5), runif(1,1.4,2.5),runif(1,1.4,2.5), runif(1,1.4,2.5),runif(1,1.4,2.5),runif(1,1.4,2.5),runif(1,1.4,2.5),runif(1,1.4,2.5),runif(1,1.4,2.5), runif(1,1.4,2.5))

eighteenb1=c(runif(1,-4.2,0),runif(1,-4.2,0),runif(1,-4.2,0),runif(1,-4.2,0),runif(1,-4.2,0),runif(1,-4.2,0), runif(1,-4.2,0), runif(1,-4.2,0), runif(1,-4.2,0), runif(1,-4.2,0), runif(1,-4.2,0), runif(1,-4.2,0), runif(1,-4.2,0), runif(1,-4.2,0), runif(1,-4.2,0), runif(1,-4.2,0), runif(1,-4.2,0), runif(1,-4.2,0), runif(1,-4.2,0))

eighteenb2= eighteenb1+ c(runif(1,1.4,2.5), runif(1,1.4,2.5), runif(1,1.4,2.5), runif(1,1.4,2.5), runif(1,1.4,2.5), runif(1,1.4,2.5), runif(1,1.4,2.5), runif(1,1.4,2.5),runif(1,1.4,2.5), runif(1,1.4,2.5), runif(1,1.4,2.5),runif(1,1.4,2.5), runif(1,1.4,2.5),runif(1,1.4,2.5),runif(1,1.4,2.5),runif(1,1.4,2.5),runif(1,1.4,2.5),runif(1,1.4,2.5), runif(1,1.4,2.5))

eighteenb3= eighteenb2+ c(runif(1,1.4,2.5), runif(1,1.4,2.5), runif(1,1.4,2.5), runif(1,1.4,2.5), runif(1,1.4,2.5), runif(1,1.4,2.5), runif(1,1.4,2.5), runif(1,1.4,2.5),runif(1,1.4,2.5), runif(1,1.4,2.5), runif(1,1.4,2.5),runif(1,1.4,2.5), runif(1,1.4,2.5),runif(1,1.4,2.5),runif(1,1.4,2.5),runif(1,1.4,2.5),runif(1,1.4,2.5),runif(1,1.4,2.5), runif(1,1.4,2.5))

eighteenb4= eighteenb3+ c(runif(1,1.4,2.5), runif(1,1.4,2.5), runif(1,1.4,2.5), runif(1,1.4,2.5), runif(1,1.4,2.5), runif(1,1.4,2.5), runif(1,1.4,2.5), runif(1,1.4,2.5),runif(1,1.4,2.5), runif(1,1.4,2.5), runif(1,1.4,2.5),runif(1,1.4,2.5), runif(1,1.4,2.5),runif(1,1.4,2.5),runif(1,1.4,2.5),runif(1,1.4,2.5),runif(1,1.4,2.5),runif(1,1.4,2.5), runif(1,1.4,2.5))

# Estimating the single-item reliability with method CA

myalpha <- function(data) {

varcovar <- var(data)

J <- length(data)

JJ <- J/(J-1)

JJ* (1 - (sum(diag(varcovar))/sum(varcovar)))

}

singlecoratt <- function(data) {

data <- na.omit(data)

J <- ncol(data)

singlerel <- matrix(NA, J)

for (j in 1:J) {

X <- myalpha(data[,-j])

Y <- cor(apply(data[,-j],1,sum),data[,j])

singlerel[j] <- (Y/sqrt(X))^2

}

return(singlerel)

}

# method λ6

L6 <- function(data) {

nummis <- sum(is.na(data))

data <- na.omit(data)

eps <- 1/diag(solve(var(data)))

var <- apply(data,2,var)

L6 <- 1 - (eps/var)

return(L6)

}

# Estimate the single-item reliability with method FA

singlepafh2 <- function(data) {

data <- na.omit(data)

J <- ncol(data)

singlerelia <- matrix(NA, J)

singlerelia <- fa(data,nfactors=1,fm="pa")$communality

return(singlerelia)

}

# estimate the single-item reliability with method LCM

compute.PP.LCM <- function(X) {

N <- nrow(X)

J <- ncol(X)

m <- max(X)+1

P <- matrix(t(apply(outer(as.matrix(X), 1:(m - 1), ">=") * 1, c(2, 3), mean)), nrow = (m - 1) * J)

label <- as.vector(t(outer(paste("P(X", 1:J, ">=", sep = ""), paste(1:(m - 1), ")", sep = ""), paste, sep = "")))

PP <- matrix(0, J * (m - 1), J * (m - 1))

i <- 0

j <- 0

for (i in 1:(J - 1)) for (j in (i + 1):J){

PP[((i - 1) * (m - 1) + 1):((i - 1) * (m - 1) + (m - 1)), ((j - 1) * (m - 1) + 1):((j - 1) * (m - 1) + (m - 1))] <- t(outer(X[,i], 0:(m - 2), ">")) %*% outer(X[, j], 0:(m - 2),">")/N

}

PP <- PP + t(PP) + kronecker(diag(J), matrix(-1, m - 1, m - 1))

PP[PP < -0.5] <- NA

dimnames(PP) <- list(label, label)

return(list(PP=PP,P=P))

}

# Check if all categories are present

check.probs <- function(L,m){

for (j in 1:length(L)){

if(ncol(L[[j]])!=m){

new.L <- NULL

cats.obs <- apply(outer(1:m,as.numeric(substr(dimnames(L[[j]])[[2]],4,4)),"=="),1,any)

for(g in 1:m){

if (cats.obs[g]){

new.L <- cbind(new.L,matrix(L[[j]][,g]))

}else{

new.L <- cbind(new.L,matrix(0,nrow=nrow(L[[j]]),ncol=1))

}

}

L[[j]] <- new.L

}

}

return(L)

}

singlelcm <- function(data) {

library(poLCA)

library(MASS)

nummis <- sum(is.na(data))

data <- na.omit(data)

data.0 <- data - min(data)

m <- max(data.0)+1

P.tmp <- compute.PP.LCM(data.0)

PP <- P.tmp$PP

P <- P.tmp$P

data.lc <- as.data.frame(data.0)+1

J <- ncol(data.lc)

names(data.lc) <- paste("V",1:J,sep="")

f <- as.formula(paste("cbind(",paste("V", 1:(J-1) , "," , sep="",collapse=""), paste("V",J,sep=""),") ~ 1", collapse=""))

AIC3.old <- 1e100

n.class <- 2

output <- list()

# Latent class estimation

repeat{

model.lc <- poLCA(f, data.lc, nclass = n.class, maxiter = 100000, nrep = 3, verbose = F)

AIC3.new <- (-2) * model.lc$llik + 3 * model.lc$npar

n.class <- n.class+1

if(AIC3.new > AIC3.old) break

AIC3.old <- AIC3.new

}

K <- n.class-1 # final number of classes

model.lc <- poLCA(f, data.lc, nclass=K, maxiter = 100000, verbose=F)

# Check whether all categories occur

probs <- check.probs(model.lc$probs,m)

pj.k <- list()

for (k in 1:K) pj.k[[k]] <- matrix(unlist(probs),nrow=K)[k,]

# pij.k bivariate probabilities given class membership implied by the LCM

pij.k <- lapply(pj.k,function(x) outer(x,x))

# pij bivariate probabilities implied by the LCM

pij <- 0

for (k in 1:K) pij <- pij + model.lc$P[k] * pij.k[[k]]

# Pij cumulative bivariate probabilities implied by the LCM

Pij <- matrix(0, J * (m - 1), J * (m - 1))

for (i in 1:J) for (j in 1:J){

pij.tmp <- pij[((i - 1) * (m) + 1):((i - 1) * (m) + (m)), ((j - 1) * (m) + 1):((j - 1) * (m) + (m))]

Pij.tmp <- matrix(NA,m,m)

for (u in 1:m) for (v in 1:m) Pij.tmp[u,v] <- sum(pij.tmp[row(pij.tmp) >= u & col(pij.tmp)>= v])

Pij[((i - 1) * (m - 1) + 1):((i - 1) * (m - 1) + (m - 1)), ((j - 1) * (m - 1) + 1):((j - 1) * (m - 1) + (m - 1))] <- Pij.tmp[2:m,2:m]

}

PP[is.na(PP)] <- Pij[is.na(PP)]

num <- PP- outer(as.numeric(P),as.numeric(P))

# Calculate the univariate probabilities

univar <- matrix(rep(NA, (m*J)), m, J)

for (j in 1:J) {

for (u in 1:m) {

som <- 0

for (k in 1:K) {

som <- som + (model.lc$P[k] * probs[[j]][k,u])

}

univar[u,j] <- som

}

}

# Calculate the item variances for the denominator

itemvar <- array(NA, dim = c(m,2,J))

for (j in 1:J) {

for (u in 1:m){

itemvar[u,1,j] <- u^2*univar[u,j]

itemvar[u,2,j] <- u*univar[u,j]

}

}

itemvari <- matrix(NA, nrow = J)

for (i in 1:J) {

itemvari[i] <- sum(itemvar[,1,i]) - (sum(itemvar[,2,i]))^2

}

# Calculate the reliability for the different items

SingleLCM <- matrix(0, nrow = J)

for (j in 1:J) {

singleLCM[j] <- sum(num[((j*(m-1))-(m-2)):(j*(m-1)),((j*(m-1))-(m-2)):(j*(m-1))])/itemvari[j]

}

output$singleLCM <- singleLCM

output$niter <- model.lc$numiter

output$time <- model.lc$time

output$nclass <- K

output$classprob <- model.lc$P

output$nummis <- nummis

return(output)

}

# Estimate the single-item reliability with method DMM

compute.PP <- function(X,P,N,J,m){

label <- as.vector(t(outer(paste("P(X",substr(as.character(100+(1:J)),2,3),">=",sep=""),paste(1:(m-1),")",sep=""), paste, sep="")))

# substr(as.character(10000+r),2,5)

PP <- matrix(0,J*(m-1),J*(m-1))

i <- 0

j <- 0

for(i in 1:(J-1)) for(j in (i+1):J)

PP[((i-1)*(m-1)+1):((i-1)*(m-1)+(m-1)),((j-1)*(m-1)+1):((j-1)*(m-1)+(m-1))] <- t(outer(X[,i],0:(m-2),">")) %*% outer(X[,j],0:(m-2),">")/N

PP <- PP + t(PP) + kronecker(diag(J),matrix(-1,m-1,m-1))

PP[PP < -.5] <- NA

dimnames(PP) <- list(label,label)

PP <- PP[order(P),order(P)]

return(PP)

}

singledmm <- function(data) {

data <- na.omit(data)

data.0 <- data - min(data)

J <- ncol(data)

m <- max(data.0) + 1

N <- nrow(data)

P <- matrix(t(apply(outer(as.matrix(data.0), 1:(m-1), ">=")*1,c(2,3),mean, na.rm=TRUE)),nrow=(m-1)*J)

PP <- compute.PP(data.0,P,N,J,m)

P <- matrix(sort(P))

off.boundary <- P > 0 & P < 1

P <- matrix(P[off.boundary,1])

PP <- PP[off.boundary,off.boundary]

km <- length(P)

is.na.PP <- is.na(PP)

lower.bound.PP <- P %*% t(P)

upper.bound.PP <- outer(as.numeric(P),as.numeric(P),FUN = "pmin")

dimnames(P) <- list(dimnames(PP)[[1]],"")

OO <- is.na(PP)

set.matrix <- outer(as.numeric(P),as.numeric(P),"==")*1

unique.cells <- which(apply(set.matrix,1,sum)==1)

Type <- set.matrix

Type[unique.cells,unique.cells] <- 0

set.vector <- sign(apply(Type,1,sum))

Type <- outer(set.vector,set.vector) + outer(rep(1,km),set.vector)*2 + outer(set.vector,rep(1,km))

for (i in 1:km) for (j in 1:km) if(is.na(PP[i,j])){

if (Type[i,j]==4) PP[i,j] <-

mean(PP[set.matrix[i,]==1,set.matrix[,j]==1],na.rm=T)

else{

if(Type[i,j]==1){

RightN <- ifelse(any(!is.na(PP[i,j:km])), j + min(which(!is.na(PP[i,j:km]))) - 1, NA)

RightPP <- ifelse(is.na(RightN),NA,mean(PP[set.matrix[i,]==1,set.matrix[,RightN]==1],na.rm=T))

LeftN <- ifelse(any(!is.na(PP[i,1:j])), max(which(!is.na(PP[i,1:j]))),NA)

LeftPP <- ifelse(is.na(LeftN),NA,mean(PP[set.matrix[i,]==1,set.matrix[,LeftN]==1],na.rm=T))

LowerN <- UpperN <- i

LowerPP <- UpperPP <- mean(PP[set.matrix[i,]==1,set.matrix[,j]==1],na.rm=T)

}

if(Type[i,j]==2){

LowerN <- ifelse(any(!is.na(PP[i:km,j])), i + min(which(!is.na(PP[i:km,j]))) - 1, NA)

LowerPP <- ifelse(is.na(LowerN),NA,mean(PP[set.matrix[LowerN,]==1,set.matrix[,j]==1],na.rm=T))

UpperN <- ifelse(any(!is.na(PP[1:i,j])), max(which(!is.na(PP[1:i,j]))),NA)

UpperPP <- ifelse(is.na(UpperN),NA,mean(PP[set.matrix[UpperN,]==1,set.matrix[,j]==1],na.rm=T))

RightN <- LeftN <- j

RightPP <- LeftPP <- mean(PP[set.matrix[i,]==1,set.matrix[,j]==1],na.rm=T)

}

if(Type[i,j]==0){

RightN <- ifelse(any(!is.na(PP[i,j:km])), j + min(which(!is.na(PP[i,j:km]))) - 1, NA)

RightPP <- ifelse(is.na(RightN),NA,mean(PP[set.matrix[i,]==1,set.matrix[,RightN]==1],na.rm=T))

LeftN <- ifelse(any(!is.na(PP[i,1:j])), max(which(!is.na(PP[i,1:j]))),NA)

LeftPP <- ifelse(is.na(LeftN),NA,mean(PP[set.matrix[i,]==1,set.matrix[,LeftN]==1],na.rm=T))

LowerN <- ifelse(any(!is.na(PP[i:km,j])), i + min(which(!is.na(PP[i:km,j]))) - 1, NA)

LowerPP <- ifelse(is.na(LowerN),NA,mean(PP[set.matrix[LowerN,]==1,set.matrix[,j]==1],na.rm=T))

UpperN <- ifelse(any(!is.na(PP[1:i,j])), max(which(!is.na(PP[1:i,j]))),NA)

UpperPP <- ifelse(is.na(UpperN),NA,mean(PP[set.matrix[UpperN,]==1,set.matrix[,j]==1],na.rm=T))

}

E17a <- ifelse(is.na(LowerN),NA,LowerPP * P[i]/P[LowerN])

E17b <- ifelse(is.na(RightN),NA,RightPP * P[j]/P[RightN])

E17c <- ifelse(is.na(UpperN),NA,UpperPP * P[i]/P[UpperN])

E17d <- ifelse(is.na(LeftN),NA, LeftPP * P[j]/P[LeftN] )

E21a <- ifelse(is.na(LowerN),NA,LowerPP * (1 - P[i])/(1 - P[LowerN]) -P[j] * (P[LowerN] - P[i])/(1 - P[LowerN]))

E21b <- ifelse(is.na(RightN),NA,RightPP * (1 - P[j])/(1 - P[RightN]) -P[i] * (P[RightN] - P[j])/(1 - P[RightN]))

E21c <- ifelse(is.na(UpperN),NA,UpperPP * (1 - P[i])/(1 - P[UpperN]) +P[j] * (P[i] - P[UpperN])/(1 - P[UpperN]))

E21d <- ifelse(is.na(LeftN) ,NA,LeftPP * (1 - P[j])/(1 - P[LeftN] ) +P[i] * (P[j] - P[LeftN])/(1 - P[LeftN] ))

PP[i,j] <- mean(c(E17a,E17b,E17c,E17d,E21a,E21b,E21c,E21d),na.rm=T)

}

}

PP[is.nan(PP)] <- 0

PP[PP > upper.bound.PP & OO] <- upper.bound.PP[PP > upper.bound.PP & OO]

PP[PP < lower.bound.PP & OO] <- lower.bound.PP[PP < lower.bound.PP & OO]

PP <- PP[sort(colnames(PP)),sort(colnames(PP))]

P <- matrix(P[order(dimnames(P)[[1]])])

rownames(P) <- dimnames(PP)[[1]]

num <- PP- outer(as.numeric(P),as.numeric(P))

find.cats <- function(x , range = -1000 : 1000) range[range %in% x]

which.cat <- list()

for (j in 1:J) {which.cat[[j]] <- find.cats(data[,j])}

number.ISRFs <- sapply(which.cat, length) - 1

position <- c(1, cumsum(number.ISRFs)) # c(1, cumsum(number.ISRFs[-1]))

position <- position[-1]

item.vars <- apply(data, 2, var)

singleLCM <- rep(NA, J)

for (j in 1 : J) {

rows <- ((position[j] - (number.ISRFs[j] - 1)) : position[j])

singleLCM[j] <- sum(num[rows, rows])/item.vars[j]

}

return(singleLCM)

}

# Calculating the single-item reliability coefficients with five methods

rsim <-1000

ndesign <-36

design <- list()

design[[1]] <- list(Q=2, rho=matrix(c(1,.65,.65,1), ncol = 2), psi=matrix(c(rep(1,6),rep(0,7), runif(1,0.4,2.8)),ncol=2), delta=cbind(sixb1,sixb2,sixb3,sixb4),J=7, N=400, m=4)

design[[2]] <- list(Q=2, rho=matrix(c(1,.65,.65,1), ncol = 2), psi=matrix(c(rep(1,12),rep(0,13), runif(1,0.4,2.8)),ncol=2), delta=cbind(twelveb1,twelveb2,twelveb3,twelveb4),J=13, N=400, m=4)

design[[3]] <- list(Q=2, rho=matrix(c(1,.65,.65,1), ncol = 2), psi=matrix(c(rep(1,18),rep(0,19), runif(1,0.4,2.8)),ncol=2), delta=cbind(eighteenb1, eighteenb2, eighteenb3, eighteenb4),J=19, N=400, m=4)

design[[4]] <- list(Q=2, rho=matrix(c(1,.65,.65,1), ncol = 2), psi=matrix(c(runif(1,0.4,2.8), runif(1,0.4,2.8), runif(1,0.4,2.8), runif(1,0.4,2.8), runif(1,0.4,2.8), runif(1,0.4,2.8), rep(0,7), runif(1,0.4,2.8)),ncol=2),

delta=cbind(sixb1,sixb2,sixb3,sixb4),J=7, N=400, m=4)

design[[5]] <- list(Q=2, rho=matrix(c(1,.65,.65,1), ncol = 2), psi=matrix(c(runif(1,0.4,2.8), runif(1,0.4,2.8), runif(1,0.4,2.8), runif(1,0.4,2.8), runif(1,0.4,2.8), runif(1,0.4,2.8), runif(1,0.4,2.8), runif(1,0.4,2.8), runif(1,0.4,2.8), runif(1,0.4,2.8), runif(1,0.4,2.8), runif(1,0.4,2.8), rep(0,13), runif(1,0.4,2.8)),ncol=2), delta=cbind(twelveb1,twelveb2,twelveb3,twelveb4),J=13, N=400, m=4)

design[[6]] <- list(Q=2, rho=matrix(c(1,.65,.65,1), ncol = 2), psi=matrix(c(runif(1,0.4,2.8), runif(1,0.4,2.8), runif(1,0.4,2.8), runif(1,0.4,2.8), runif(1,0.4,2.8), runif(1,0.4,2.8), runif(1,0.4,2.8), runif(1,0.4,2.8), runif(1,0.4,2.8), runif(1,0.4,2.8), runif(1,0.4,2.8), runif(1,0.4,2.8), runif(1,0.4,2.8), runif(1,0.4,2.8), runif(1,0.4,2.8), runif(1,0.4,2.8), runif(1,0.4,2.8), runif(1,0.4,2.8),rep(0,19), runif(1,0.4,2.8)),ncol=2), delta=cbind(eighteenb1, eighteenb2, eighteenb3, eighteenb4),J=19, N=400, m=4)

design[[7]] <- list(Q=2, rho=matrix(c(1,.75,.75,1), ncol = 2), psi=matrix(c(rep(1,6),rep(0,7), runif(1,0.4,2.8)),ncol=2), delta=cbind(sixb1,sixb2,sixb3,sixb4),J=7, N=400, m=4)

design[[8]] <- list(Q=2, rho=matrix(c(1,.75,.75,1), ncol = 2), psi=matrix(c(rep(1,12),rep(0,13), runif(1,0.4,2.8)),ncol=2), delta=cbind(twelveb1,twelveb2,twelveb3,twelveb4),J=13, N=400, m=4)

design[[9]] <- list(Q=2, rho=matrix(c(1,.75,.75,1), ncol = 2), psi=matrix(c(rep(1,18),rep(0,19), runif(1,0.4,2.8)),ncol=2), delta=cbind(eighteenb1, eighteenb2, eighteenb3, eighteenb4),J=19, N=400, m=4)

design[[10]] <- list(Q=2, rho=matrix(c(1,.75,.75,1), ncol = 2), psi=matrix(c(runif(1,0.4,2.8), runif(1,0.4,2.8), runif(1,0.4,2.8), runif(1,0.4,2.8), runif(1,0.4,2.8), runif(1,0.4,2.8), rep(0,7), runif(1,0.4,2.8)),ncol=2),

delta=cbind(sixb1,sixb2,sixb3,sixb4),J=7, N=400, m=4)

design[[11]] <- list(Q=2, rho=matrix(c(1,.75,.75,1), ncol = 2), psi=matrix(c(runif(1,0.4,2.8), runif(1,0.4,2.8), runif(1,0.4,2.8), runif(1,0.4,2.8), runif(1,0.4,2.8), runif(1,0.4,2.8), runif(1,0.4,2.8), runif(1,0.4,2.8), runif(1,0.4,2.8), runif(1,0.4,2.8), runif(1,0.4,2.8), runif(1,0.4,2.8), rep(0,13), runif(1,0.4,2.8)),ncol=2), delta=cbind(twelveb1,twelveb2,twelveb3,twelveb4),J=13, N=400, m=4)

design[[12]] <- list(Q=2, rho=matrix(c(1,.75,.75,1), ncol = 2), psi=matrix(c(runif(1,0.4,2.8), runif(1,0.4,2.8), runif(1,0.4,2.8), runif(1,0.4,2.8), runif(1,0.4,2.8), runif(1,0.4,2.8), runif(1,0.4,2.8), runif(1,0.4,2.8), runif(1,0.4,2.8), runif(1,0.4,2.8), runif(1,0.4,2.8), runif(1,0.4,2.8), runif(1,0.4,2.8), runif(1,0.4,2.8), runif(1,0.4,2.8), runif(1,0.4,2.8), runif(1,0.4,2.8), runif(1,0.4,2.8),rep(0,19), runif(1,0.4,2.8)),ncol=2), delta=cbind(eighteenb1, eighteenb2, eighteenb3, eighteenb4),J=19, N=400, m=4)

design[[13]] <- list(Q=2, rho=matrix(c(1,.85,.85,1), ncol = 2), psi=matrix(c(rep(1,6),rep(0,7), runif(1,0.4,2.8)),ncol=2), delta=cbind(sixb1,sixb2,sixb3,sixb4),J=7, N=400, m=4)

design[[14]] <- list(Q=2, rho=matrix(c(1,.85,.85,1), ncol = 2), psi=matrix(c(rep(1,12),rep(0,13), runif(1,0.4,2.8)),ncol=2), delta=cbind(twelveb1,twelveb2,twelveb3,twelveb4),J=13, N=400, m=4)

design[[15]] <- list(Q=2, rho=matrix(c(1,.85,.85,1), ncol = 2), psi=matrix(c(rep(1,18),rep(0,19), runif(1,0.4,2.8)),ncol=2), delta=cbind(eighteenb1, eighteenb2, eighteenb3, eighteenb4),J=19, N=400, m=4)

design[[16]] <- list(Q=2, rho=matrix(c(1,.85,.85,1), ncol = 2), psi=matrix(c(runif(1,0.4,2.8), runif(1,0.4,2.8), runif(1,0.4,2.8), runif(1,0.4,2.8), runif(1,0.4,2.8), runif(1,0.4,2.8), rep(0,7), runif(1,0.4,2.8)),ncol=2),

delta=cbind(sixb1,sixb2,sixb3,sixb4),J=7, N=400, m=4)

design[[17]] <- list(Q=2, rho=matrix(c(1,.85,.85,1), ncol = 2), psi=matrix(c(runif(1,0.4,2.8), runif(1,0.4,2.8), runif(1,0.4,2.8), runif(1,0.4,2.8), runif(1,0.4,2.8), runif(1,0.4,2.8), runif(1,0.4,2.8), runif(1,0.4,2.8), runif(1,0.4,2.8), runif(1,0.4,2.8), runif(1,0.4,2.8), runif(1,0.4,2.8), rep(0,13), runif(1,0.4,2.8)),ncol=2), delta=cbind(twelveb1,twelveb2,twelveb3,twelveb4),J=13, N=400, m=4)

design[[18]] <- list(Q=2, rho=matrix(c(1,.85,.85,1), ncol = 2), psi=matrix(c(runif(1,0.4,2.8), runif(1,0.4,2.8), runif(1,0.4,2.8), runif(1,0.4,2.8), runif(1,0.4,2.8), runif(1,0.4,2.8), runif(1,0.4,2.8), runif(1,0.4,2.8), runif(1,0.4,2.8), runif(1,0.4,2.8), runif(1,0.4,2.8), runif(1,0.4,2.8), runif(1,0.4,2.8), runif(1,0.4,2.8), runif(1,0.4,2.8), runif(1,0.4,2.8), runif(1,0.4,2.8), runif(1,0.4,2.8),rep(0,19), runif(1,0.4,2.8)),ncol=2), delta=cbind(eighteenb1, eighteenb2, eighteenb3, eighteenb4),J=19, N=400, m=4)

design[[19]] <- list(Q=2, rho=matrix(c(1,.65,.65,1), ncol = 2), psi=matrix(c(rep(1,6),rep(0,7), runif(1,0.4,2.8)),ncol=2), delta=cbind(sixb1,sixb2,sixb3,sixb4),J=7, N=1000, m=4)

design[[20]] <- list(Q=2, rho=matrix(c(1,.65,.65,1), ncol = 2), psi=matrix(c(rep(1,12),rep(0,13), runif(1,0.4,2.8)),ncol=2), delta=cbind(twelveb1,twelveb2,twelveb3,twelveb4),J=13, N=1000, m=4)

design[[21]] <- list(Q=2, rho=matrix(c(1,.65,.65,1), ncol = 2), psi=matrix(c(rep(1,18),rep(0,19), runif(1,0.4,2.8)),ncol=2), delta=cbind(eighteenb1, eighteenb2, eighteenb3, eighteenb4),J=19, N=1000, m=4)

design[[22]] <- list(Q=2, rho=matrix(c(1,.65,.65,1), ncol = 2), psi=matrix(c(runif(1,0.4,2.8), runif(1,0.4,2.8), runif(1,0.4,2.8), runif(1,0.4,2.8), runif(1,0.4,2.8), runif(1,0.4,2.8), rep(0,7), runif(1,0.4,2.8)),ncol=2),

delta=cbind(sixb1,sixb2,sixb3,sixb4),J=7, N=1000, m=4)

design[[23]] <- list(Q=2, rho=matrix(c(1,.65,.65,1), ncol = 2), psi=matrix(c(runif(1,0.4,2.8), runif(1,0.4,2.8), runif(1,0.4,2.8), runif(1,0.4,2.8), runif(1,0.4,2.8), runif(1,0.4,2.8), runif(1,0.4,2.8), runif(1,0.4,2.8), runif(1,0.4,2.8), runif(1,0.4,2.8), runif(1,0.4,2.8), runif(1,0.4,2.8), rep(0,13), runif(1,0.4,2.8)),ncol=2), delta=cbind(twelveb1,twelveb2,twelveb3,twelveb4),J=13, N=1000, m=4)

design[[24]] <- list(Q=2, rho=matrix(c(1,.65,.65,1), ncol = 2), psi=matrix(c(runif(1,0.4,2.8), runif(1,0.4,2.8), runif(1,0.4,2.8), runif(1,0.4,2.8), runif(1,0.4,2.8), runif(1,0.4,2.8), runif(1,0.4,2.8), runif(1,0.4,2.8), runif(1,0.4,2.8), runif(1,0.4,2.8), runif(1,0.4,2.8), runif(1,0.4,2.8), runif(1,0.4,2.8), runif(1,0.4,2.8), runif(1,0.4,2.8), runif(1,0.4,2.8), runif(1,0.4,2.8), runif(1,0.4,2.8),rep(0,19), runif(1,0.4,2.8)),ncol=2), delta=cbind(eighteenb1, eighteenb2, eighteenb3, eighteenb4),J=19, N=1000, m=4)

design[[25]] <- list(Q=2, rho=matrix(c(1,.75,.75,1), ncol = 2), psi=matrix(c(rep(1,6),rep(0,7), runif(1,0.4,2.8)),ncol=2), delta=cbind(sixb1,sixb2,sixb3,sixb4),J=7, N=1000, m=4)

design[[26]] <- list(Q=2, rho=matrix(c(1,.75,.75,1), ncol = 2), psi=matrix(c(rep(1,12),rep(0,13), runif(1,0.4,2.8)),ncol=2), delta=cbind(twelveb1,twelveb2,twelveb3,twelveb4),J=13, N=1000, m=4)

design[[27]] <- list(Q=2, rho=matrix(c(1,.75,.75,1), ncol = 2), psi=matrix(c(rep(1,18),rep(0,19), runif(1,0.4,2.8)),ncol=2), delta=cbind(eighteenb1, eighteenb2, eighteenb3, eighteenb4),J=19, N=1000, m=4)

design[[28]] <- list(Q=2, rho=matrix(c(1,.75,.75,1), ncol = 2), psi=matrix(c(runif(1,0.4,2.8), runif(1,0.4,2.8), runif(1,0.4,2.8), runif(1,0.4,2.8), runif(1,0.4,2.8), runif(1,0.4,2.8), rep(0,7), runif(1,0.4,2.8)),ncol=2),

delta=cbind(sixb1,sixb2,sixb3,sixb4),J=7, N=1000, m=4)

design[[29]] <- list(Q=2, rho=matrix(c(1,.75,.75,1), ncol = 2), psi=matrix(c(runif(1,0.4,2.8), runif(1,0.4,2.8), runif(1,0.4,2.8), runif(1,0.4,2.8), runif(1,0.4,2.8), runif(1,0.4,2.8), runif(1,0.4,2.8), runif(1,0.4,2.8), runif(1,0.4,2.8), runif(1,0.4,2.8), runif(1,0.4,2.8), runif(1,0.4,2.8), rep(0,13), runif(1,0.4,2.8)),ncol=2), delta=cbind(twelveb1,twelveb2,twelveb3,twelveb4),J=13, N=1000, m=4)

design[[30]] <- list(Q=2, rho=matrix(c(1,.75,.75,1), ncol = 2), psi=matrix(c(runif(1,0.4,2.8), runif(1,0.4,2.8), runif(1,0.4,2.8), runif(1,0.4,2.8), runif(1,0.4,2.8), runif(1,0.4,2.8), runif(1,0.4,2.8), runif(1,0.4,2.8), runif(1,0.4,2.8), runif(1,0.4,2.8), runif(1,0.4,2.8), runif(1,0.4,2.8), runif(1,0.4,2.8), runif(1,0.4,2.8), runif(1,0.4,2.8), runif(1,0.4,2.8), runif(1,0.4,2.8), runif(1,0.4,2.8),rep(0,19), runif(1,0.4,2.8)),ncol=2), delta=cbind(eighteenb1, eighteenb2, eighteenb3, eighteenb4),J=19, N=1000, m=4)

design[[31]] <- list(Q=2, rho=matrix(c(1,.85,.85,1), ncol = 2), psi=matrix(c(rep(1,6),rep(0,7), runif(1,0.4,2.8)),ncol=2), delta=cbind(sixb1,sixb2,sixb3,sixb4),J=7, N=1000, m=4)

design[[32]] <- list(Q=2, rho=matrix(c(1,.85,.85,1), ncol = 2), psi=matrix(c(rep(1,12),rep(0,13), runif(1,0.4,2.8)),ncol=2), delta=cbind(twelveb1,twelveb2,twelveb3,twelveb4),J=13, N=1000, m=4)

design[[33]] <- list(Q=2, rho=matrix(c(1,.85,.85,1), ncol = 2), psi=matrix(c(rep(1,18),rep(0,19), runif(1,0.4,2.8)),ncol=2), delta=cbind(eighteenb1, eighteenb2, eighteenb3, eighteenb4),J=19, N=1000, m=4)

design[[34]] <- list(Q=2, rho=matrix(c(1,.85,.85,1), ncol = 2), psi=matrix(c(runif(1,0.4,2.8), runif(1,0.4,2.8), runif(1,0.4,2.8), runif(1,0.4,2.8), runif(1,0.4,2.8), runif(1,0.4,2.8), rep(0,7), runif(1,0.4,2.8)),ncol=2),

delta=cbind(sixb1,sixb2,sixb3,sixb4),J=7, N=1000, m=4)

design[[35]] <- list(Q=2, rho=matrix(c(1,.85,.85,1), ncol = 2), psi=matrix(c(runif(1,0.4,2.8), runif(1,0.4,2.8), runif(1,0.4,2.8), runif(1,0.4,2.8), runif(1,0.4,2.8), runif(1,0.4,2.8), runif(1,0.4,2.8), runif(1,0.4,2.8), runif(1,0.4,2.8), runif(1,0.4,2.8), runif(1,0.4,2.8), runif(1,0.4,2.8), rep(0,13), runif(1,0.4,2.8)),ncol=2), delta=cbind(twelveb1,twelveb2,twelveb3,twelveb4),J=13, N=1000, m=4)

design[[36]] <- list(Q=2, rho=matrix(c(1,.85,.85,1), ncol = 2), psi=matrix(c(runif(1,0.4,2.8), runif(1,0.4,2.8), runif(1,0.4,2.8), runif(1,0.4,2.8), runif(1,0.4,2.8), runif(1,0.4,2.8), runif(1,0.4,2.8), runif(1,0.4,2.8), runif(1,0.4,2.8), runif(1,0.4,2.8), runif(1,0.4,2.8), runif(1,0.4,2.8), runif(1,0.4,2.8), runif(1,0.4,2.8), runif(1,0.4,2.8), runif(1,0.4,2.8), runif(1,0.4,2.8), runif(1,0.4,2.8),rep(0,19), runif(1,0.4,2.8)),ncol=2), delta=cbind(eighteenb1, eighteenb2, eighteenb3, eighteenb4),J=19, N=1000, m=4)

dmm <- list()

for (i in 1:ndesign) dmm[[i]] <- matrix(NA, nrow = rsim, ncol = design[[i]]$J)

lambda6 <- list()

for (i in 1:ndesign) lambda6[[i]] <- matrix(NA, nrow = rsim, ncol = design[[i]]$J)

lcm <- list()

for (i in 1:ndesign) lcm[[i]] <- matrix(NA, nrow = rsim, ncol = design[[i]]$J)

coratt <- list()

for (i in 1:ndesign) coratt[[i]] <- matrix(NA, nrow = rsim, ncol = design[[i]]$J)

factor <- list()

for (i in 1:ndesign) factor[[i]] <- matrix(NA, nrow = rsim, ncol = design[[i]]$J)

niter <- array(NA, dim = c(rsim, 1, ndesign))

nclass <- matrix(NA, nrow = rsim, ncol = ndesign)

classprob <- list()

for (i in 1:ndesign) classprob[[i]] <- list()

# Read in every datafile and calculate the single-item reliabilities

# choose the WD where you saved the datafiles

setwd("C:/scholar/graduate school/dissertation/dataset")

# setting a seed because the LCM estimations have a random component

set.seed(944507)

for (i in 1:ndesign) {

for (r in 1:rsim) {

# Read in the data

data <- read.table(paste("dataDES",i, "DATA", substr(as.character(10000+r),2,5), ".dat", sep = ""))

# Calculate the Latent Class Reliability Coefficient

LCM <- singlelcm(data)

lcm[[i]][r,] <- LCM$singleLCM

niter[r,,i] <- LCM$niter

nclass[r,i] <- LCM$nclass

classprob[[i]][[r]] <- LCM$classprob

# Calculate the DMM single-item reliability coefficient

dmm[[i]][r,] <- singledmm(data)

# Calculate lambda-6 for a single item

lambda6[[i]][r,] <- L6(data)

# Calculate the single-item reliability using the correction for attenuation method

coratt[[i]][r,] <- singlecoratt(data)

# Calculate the single-item reliability using factor analysis

factor[[i]][r,] <- singlepafh2(data)

# Show where we are in the simulation

print(paste(i,r,sep = ","))

}

save(lcm, file = "lcm.rdata")

save(dmm, file = "dmm.rdata")

save(lambda6, file = "lambda6.rdata")

save(coratt, file = "coratt.rdata")

save(factor, file = "factor.rdata")

}
